# Supplementary material for: Case of complete response to immunotherapy in MMR-deficient prostate cancer associated with NK-like and CD4+CD8+ T cells
Source: Cell Rep Med. 2026 Jun 26;7(7):102889. doi: 10.1016/j.xcrm.2026.102889 (PMC13400160; doi:10.1016/j.xcrm.2026.102889)
Supplement: Document S1. Figures S1–S6 [file mmc1.pdf]

Cell Reports Medicine, Volume 7

Supplemental information

Case of complete response to immunotherapy in MMR-deficient prostate cancer associated with NK-like and $CD4^{+}CD8^{+}$ T cells

Alexander K. Tsai, John R. Lozada, Philippa R. Kennedy, David Moline, Rachana Pandey, Riley C. Lyons, Christine Luo, Rulin Wang, Ali T. Arafa, Elise L. Femino, Sarah Zipkowitz, Alexis Figueroa, Patrick J. McCann, Matthew C. Dallos, Andrew Elliott, Paari Murugan, Martin Felices, Nicholas A. Zorko, Badrinath R. Konety, Scott M. Dehm, Jeffrey S. Miller, Steven S. Shen, Elizabeth A. Thompson, Laura A. Sena, Srinivasan Yegnasubramanian, Justin Hwang, and Emmanuel S. Antonarakis

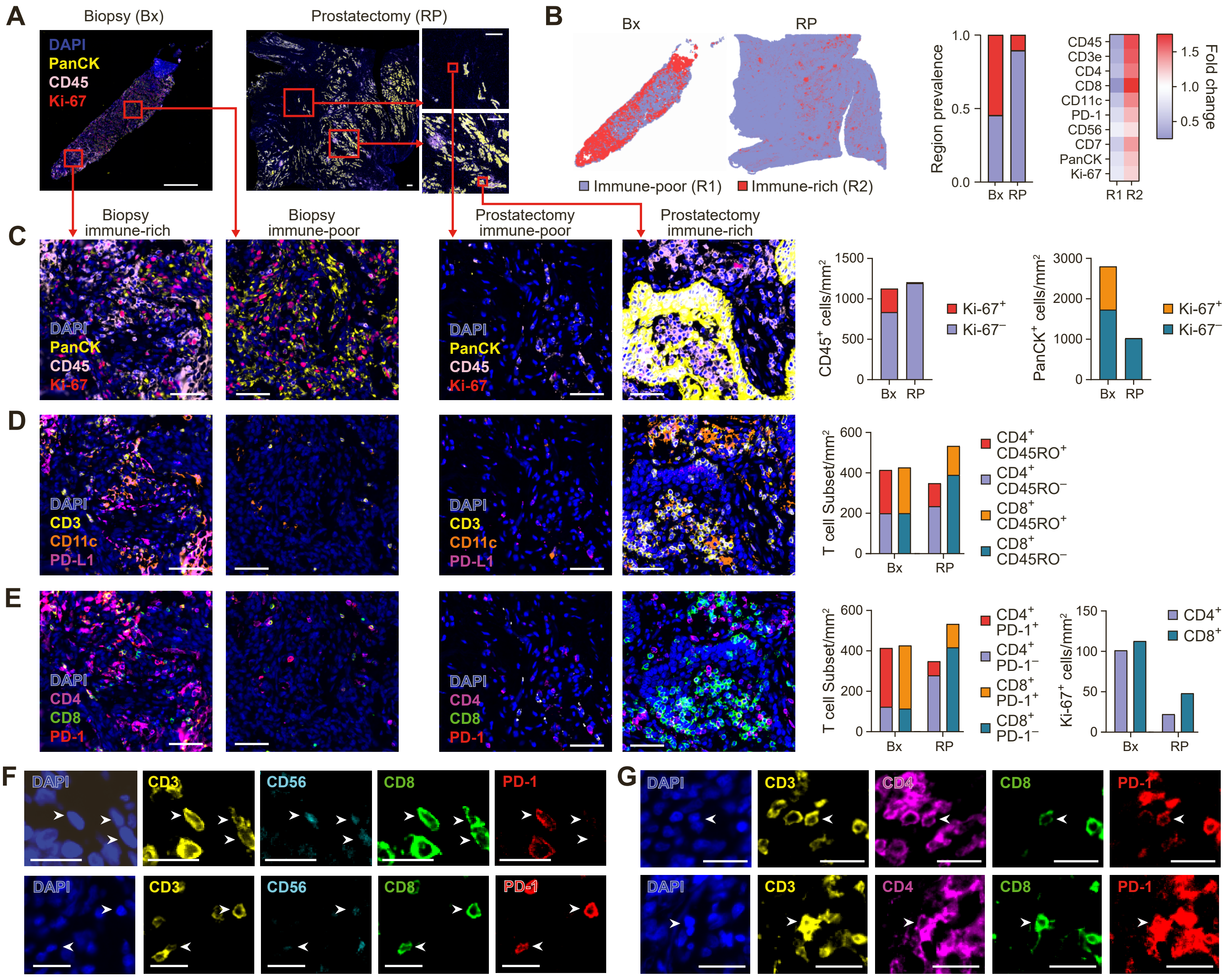

Figure S1. Antigen-experienced T cells were enriched in pre-ICI prostate cancer biopsies. Related to Figures 1 and 2. Multiplex tissue imaging of formalin-fixed, paraffin-embedded (FFPE) biopsy (Bx; pre-treatment) and prostatectomy (RP; post-ICI). All channels are scaled consistently apart from pancytokeratin (A, C) which is dimmer on tumor cells (biopsy) than glands (prostatectomy). Consequently, two different PanCK thresholds were applied to biopsy and prostatectomy samples to visualize tumor position. Quantifications of cell populations in (C-E) are shown in right panels and were performed in QuPATH.

(A) Representative channels and regions from biopsy and prostatectomy specimens. Insets show areas used for representative images in further panels. Scale bars, 0.5 mm.

(B) Unsupervised region determination by cytoMAP software depicting immune-poor (R1) and immune-rich (R2) regions in biopsies and prostatectomy tissue. Quantifications are shown in the middle panel while fold changes for individual markers between R1 and R2 regions are shown in the right panel.

(C) Representative images (left) depict PanCK$^+$ tumor cells (biopsy) and glandular cells (prostatectomy), CD45$^+$ leukocytes, and proliferating Ki-67$^+$ cells. Scale bars, 50 µm. Graphs (right) quantify Ki-67$^+$ proliferating CD45$^+$ leukocytes and PanCK$^+$ tumor or glandular cells.

(D) Representative images (left) depict CD3$^+$ T cells, CD11c$^+$ cells, and expression of PD-L1. Scale bars, 50 µm. Graph (right) quantifies CD45RO$^+$ (antigen-experienced) CD4$^+$ and CD8$^+$ T cells.

(E) Representative images (left) depict CD4$^+$ and CD8$^+$ T cells and PD-1 expression. Scale bars, 50 µm. Graphs (right) quantify PD-1$^+$ (antigen-experienced and possibly exhausted) and Ki-67$^+$ (proliferating) CD4$^+$ and CD8$^+$ T cells.

(F) Representative CD3$^+$CD8$^+$CD56$^+$ "NK-like" T cells, indicated with white arrows. Scale bars, 20 µm.

(G) Representative CD3$^+$CD4$^+$CD8$^+$ "double-positive" (DP) T cells, indicated with white arrowheads. Scale bars, 20 µm.

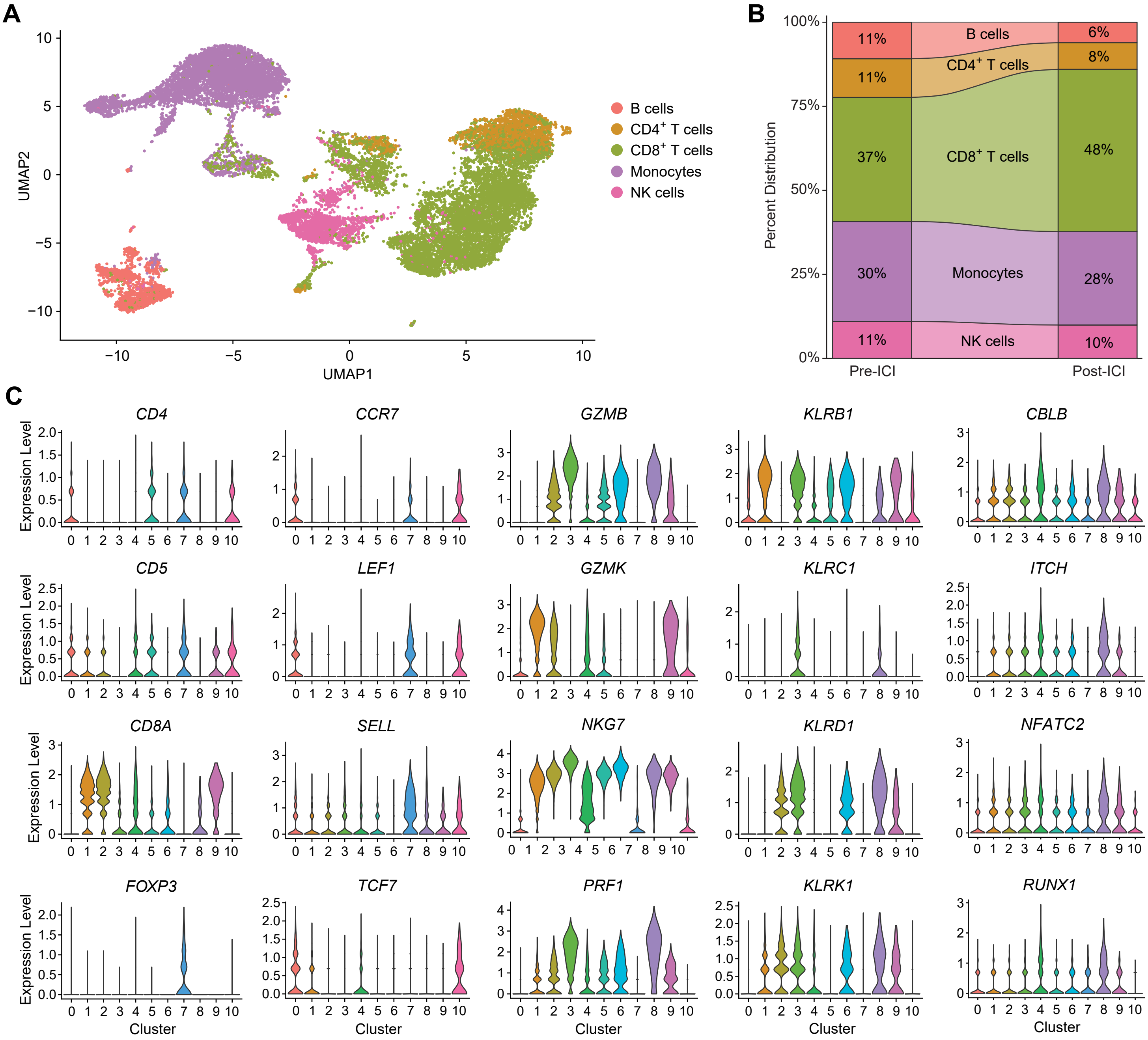

Figure S2. NK/T cell cluster annotation. Related to Figure 2.

(A) Clustering of PBMCs compiled from single-cell RNA-sequencing performed using pre-ICI and post-ICI timepoint #1 identifying B cells, CD4$^+$ T cells, CD8$^+$ T cells, monocytes, and NK cells.

(B) Distribution of cell subsets in (A) at pre-ICI and post-ICI timepoints.

(C) Expression of T cell genes (*CD4*, *CD5*, *CD8A*, *FOXP3*), memory/naïve T cells (*CCR7*, *LEF1*, *SELL*, *TCF7*), T cell effector genes (*GZMB*, *GZMK*, *NKG7*, *PRF1*), NK-associated genes (*KLRB1*, *KLRC1*, *KLRD1*, *KLRK1*), and anergy-associated genes (*CBLB*, *ITCH*, *NFATC2*, *RUNX1*) within all NK/T cell clusters (see Figure 2A).

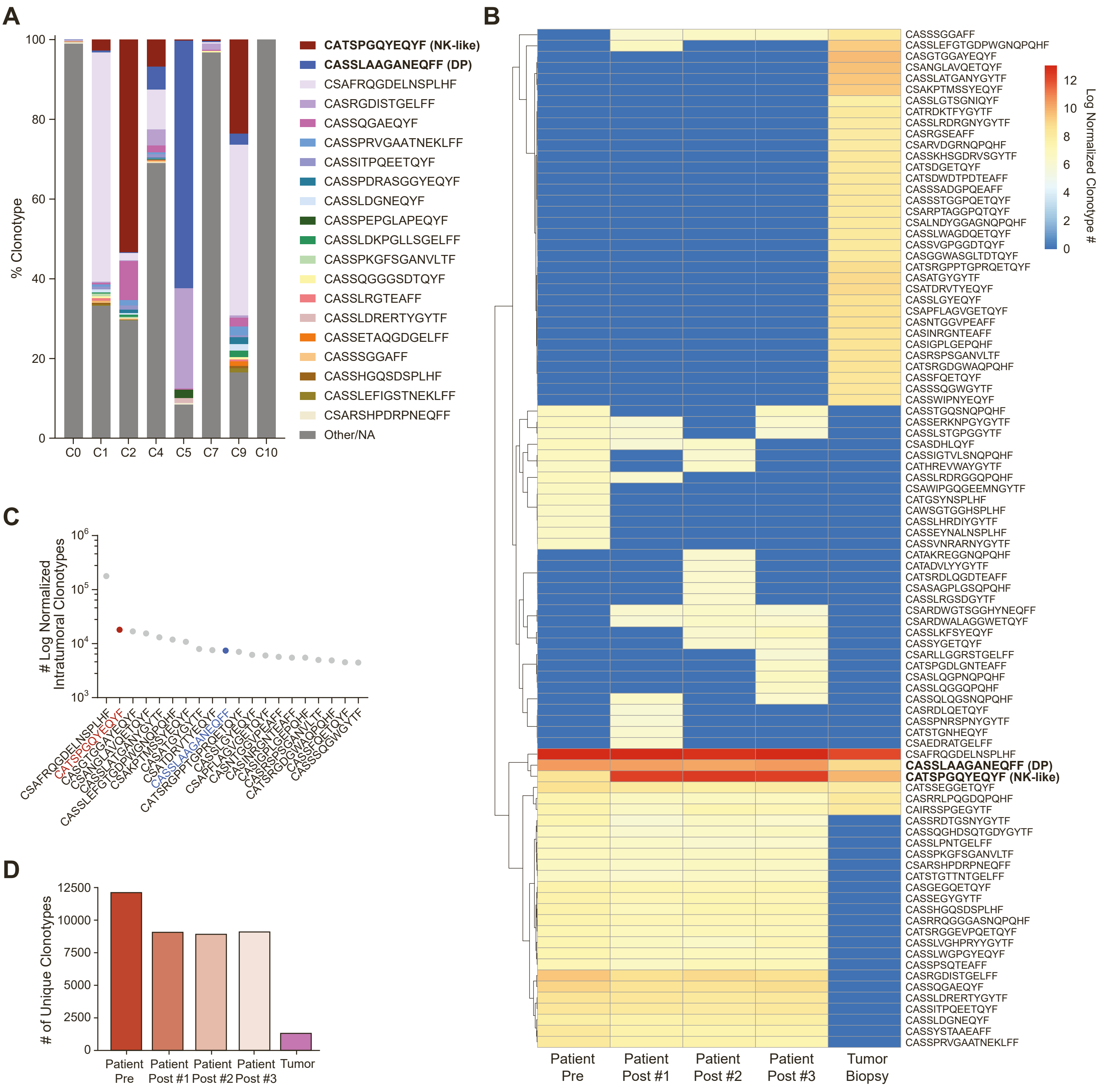

Figure S3. Intratumoral and peripheral TCR repertoire. Related to Figure 2.

(A) Frequency of the most abundant 20 TCR clonotypes within each T cell cluster for combined PBMC Pre and Post #1 timepoints as assessed by scTCR-seq. The designation "Other/NA" includes all other TCR clonotypes and cells lacking TCR data.

(B) TCR clonotypes detected within circulation and in pre-treatment tumor biopsy tissue as assessed by bulk TCR-sequencing. The top 40 most abundant log-transformed clonotypes from each of the five samples are shown after count-per-million (cpm) normalization and filtering.

(C) Log-transformed normalized clonotype count in tumor biopsy tissue by bulk TCR-sequencing.

(D) Number of unique clonotypes detected within circulation and in pre-treatment tumor biopsy tissue by bulk TCR-sequencing.

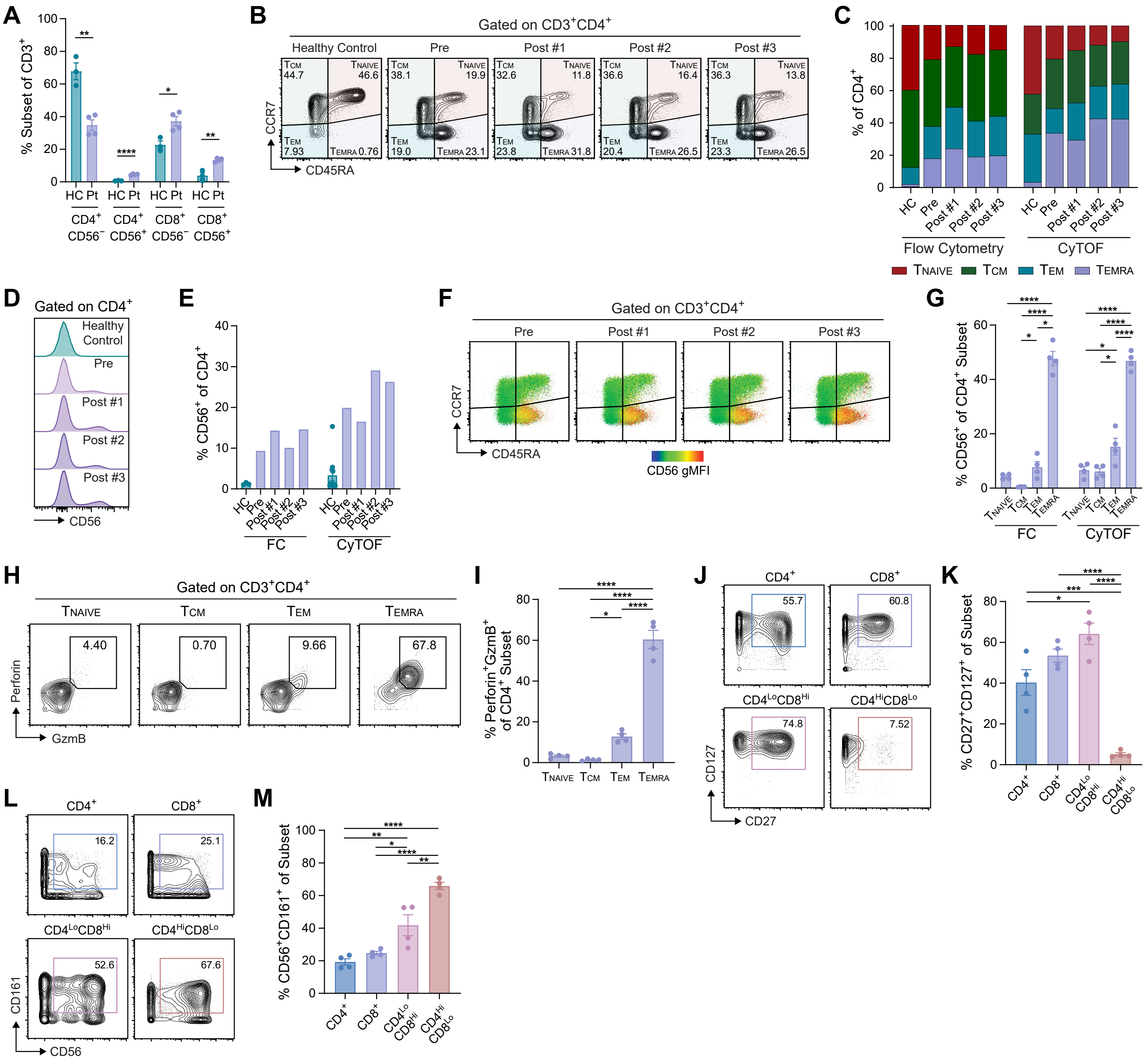

Figure S4. NK-like T cells differentiate into TEMRA and exhibited cytotoxic potential. Related to Figure 3.

(A) CD4$^+$CD56$^-$, CD4$^+$CD56$^+$, CD8$^+$CD56$^-$, and CD8$^+$CD56$^+$ frequencies within the T cell (CD45$^+$CD3$^+$) compartment of healthy control (HC) and patient PBMCs at all four timepoints assessed by flow cytometry. Unpaired t-tests were used for comparisons with statistical significance denoted as * p<0.05, ** p<0.01, and **** p<0.0001.

(B-C) Representative plots (B) of frequencies of TNAIVE (CCR7$^+$CD45RA$^+$), TCM (CCR7$^+$CD45RA$^-$), TEM (CCR7$^-$CD45RA$^-$), and TEMRA (CCR7$^-$CD45RA$^+$) within the CD4$^+$ T cell compartment of HC and patient PBMCs (C) assessed by flow cytometry and CyTOF.

(D) Histograms of CD56 on CD4$^+$ T cells from flow cytometry showing a representative HC and the patient at all four timepoints.

(E) CD56$^+$ frequencies of CD4$^+$ T cells from patient and HC PBMCs assessed by flow cytometry and CyTOF.

(F-G) Heatmaps of CD56 geometric mean fluorescent intensity (gMFI; F) with CD56$^+$ frequencies based on TNAIVE, TCM, TEM, and TEMRA CD4$^+$ T cell subsets (combined across all four timepoints; G) assessed by flow cytometry and CyTOF. ANOVA with Tukey tests were used for comparisons with statistical significance denoted as * p<0.05 and **** p<0.0001.

(H-I) Representative plots (H) of perforin$^+$granzyme-B$^+$ (GzmB$^+$) frequencies of TNAIVE, TCM, TEM, and TEMRA CD4$^+$ T cell subsets (combined across all four timepoints; I) assessed by CyTOF. ANOVA with Tukey tests were used for comparisons with statistical significance denoted as * p<0.05 and **** p<0.0001.

(J-K) Representative plots (J) of memory-associated CD27$^+$CD127$^+$ cells within CD4$^+$, CD8$^+$, CD4^{Lo}CD8^{Hi}, and CD4^{Hi}CD8^{Lo} patient-derived T cells (K) assessed by CyTOF. ANOVA with Tukey tests were used for comparisons with statistical significance denoted as * p<0.05, *** p<0.001, and **** p<0.0001.

(L-M) Representative plots (L) of NK-associated CD56$^+$CD161$^+$ cells within CD4$^+$, CD8$^+$, CD4^{Lo}CD8^{Hi}, and CD4^{Hi}CD8^{Lo} patient-derived T cells (M) assessed by CyTOF. ANOVA with Tukey tests were used for comparisons with statistical significance denoted as * p<0.05, ** p<0.01, and **** p<0.0001.

All panels show mean ± SEM.

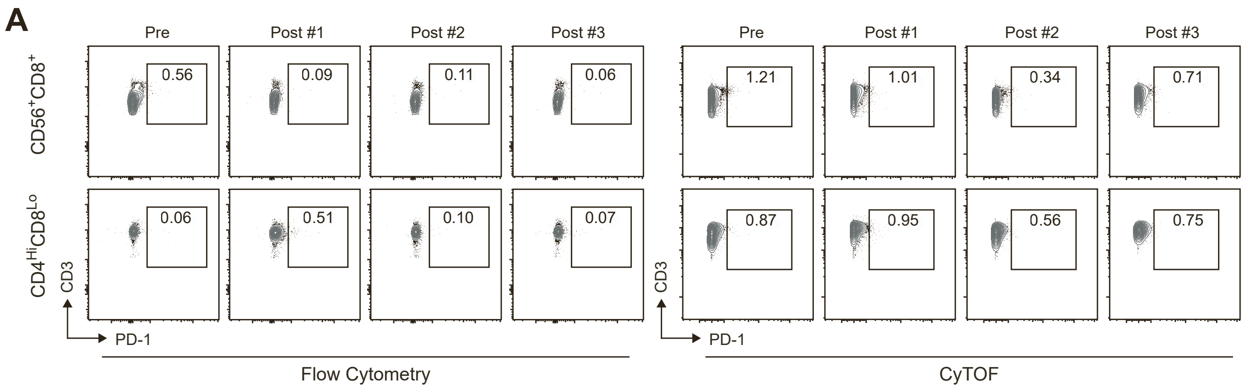

Figure S5. Circulating NK-like and DP T cells lack PD-1. Related to Figure 3.
(A) Plots from spectral flow cytometry (left) and CyTOF (right) of PD-1^{+} frequency of either CD56^{+}CD8^{+} NK-like T cells (upper) or CD4^{Hi}CD8^{Lo} DP T cells (lower) from all four PBMC timepoints.

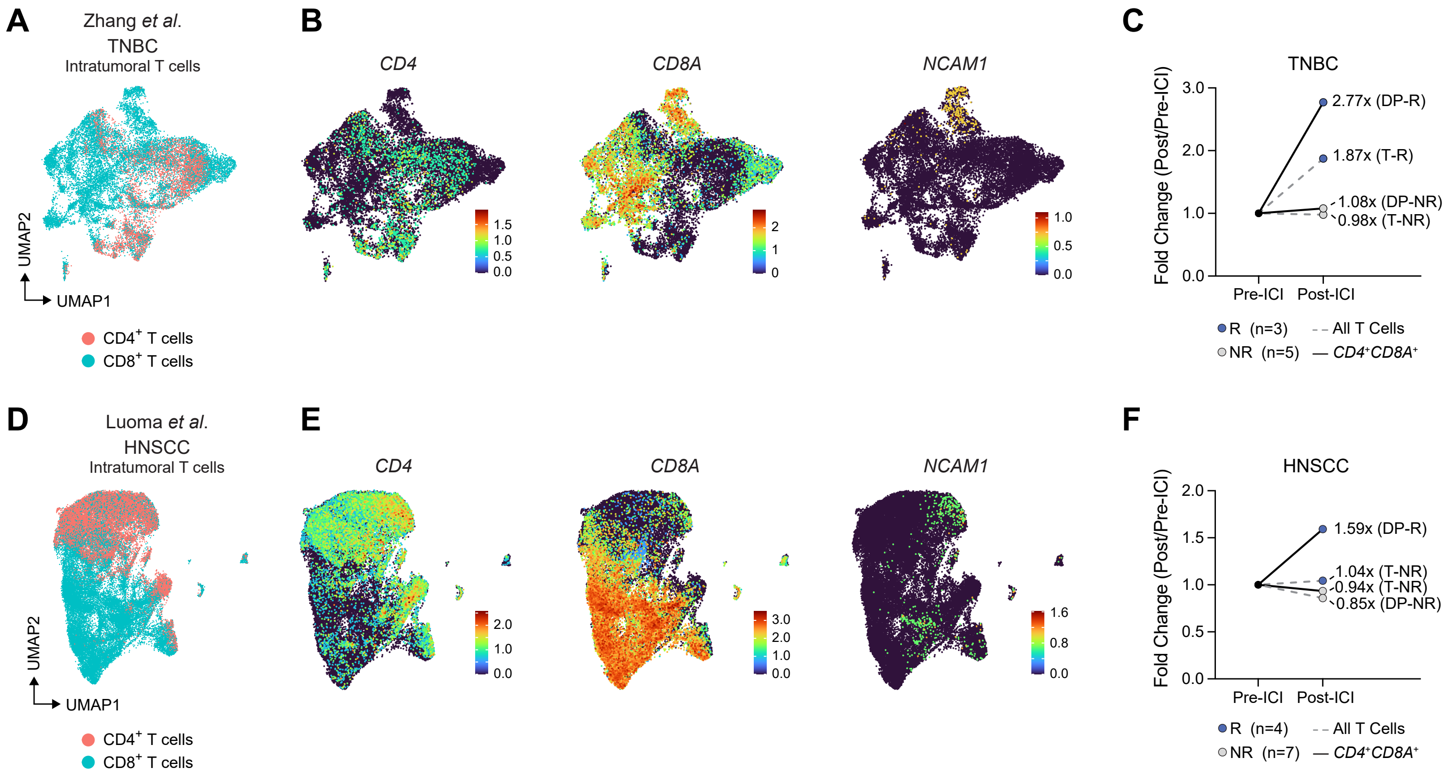

Figure S6. CD4^{+}CD8^{+} T cells expand in TNBC and HNSCC ICI responders. Related to Figure 4.
(A) T cell annotation from scRNA-seq of intratumoral T cells in triple-negative breast cancer (TNBC) patients treated with paclitaxel + atezolizumab (see reference 16).
(B) *CD4*, *CD8A*, and *NCAM1* gene expression in TNBC patients.
(C) Fold change in *CD4^{+}CD8A^{+}* double-positive T cells and all T cells segregated by responders (R) and nonresponders (NR).
(D) T cell annotation from scRNA-seq of intratumoral T cells in head/neck squamous cell carcinoma (HSNCC) patients treated with neoadjuvant nivolumab ± ipilimumab (see reference 17).
(E) *CD4*, *CD8A*, and *NCAM1* gene expression in HNSCC patients.
(F) Fold change in *CD4^{+}CD8A^{+}* double-positive T cells and all T cells segregated by responders (R) and nonresponders (NR).
