# Supplementary material for: Case of complete response to immunotherapy in MMR-deficient prostate cancer associated with NK-like and CD4+CD8+ T cells
Source: Cell Rep Med. 2026 Jun 26;7(7):102889. doi: 10.1016/j.xcrm.2026.102889 (PMC13400160; doi:10.1016/j.xcrm.2026.102889)
Supplement: Document S2. Article plus supplemental information [file mmc3.pdf]

Cell Reports
Medicine

Report

Case of complete response to immunotherapy in MMR-deficient prostate cancer associated with NK-like and $CD4^+CD8^+$ T cells

Graphical abstract

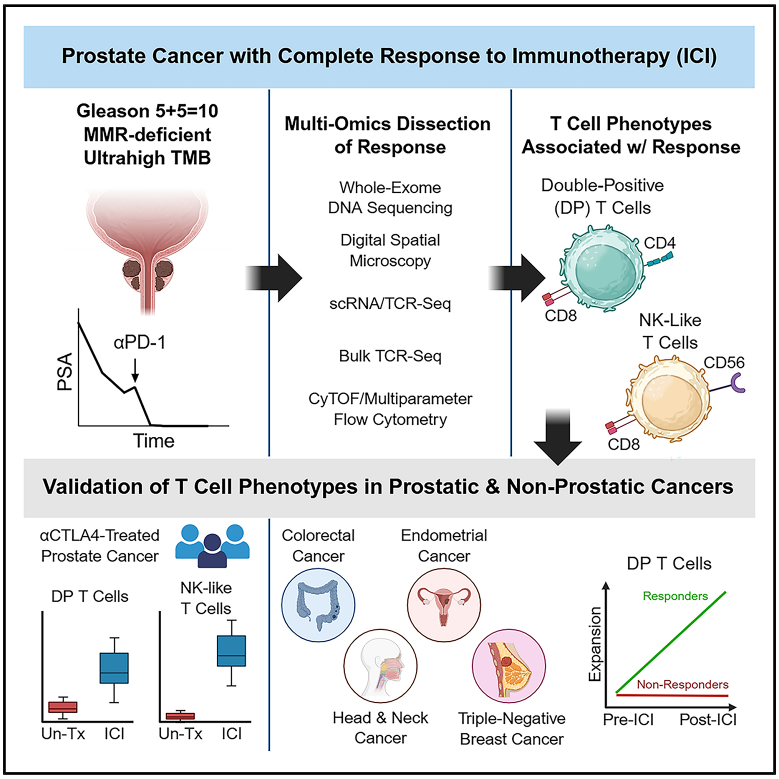

Highlights

- Pembrolizumab yielded complete response in patient with dMMR prostate cancer and ultrahigh TMB
- "NK-like" and $CD4^+CD8^+$ "double-positive" (DP) T cells expanded after immunotherapy
- NK-like and DP T cells also expanded in other cancer cohorts of immunotherapy responders

Authors

Alexander K. Tsai, John R. Lozada, Philippa R. Kennedy, ..., Srinivasan Yegnasubramanian, Justin Hwang, Emmanuel S. Antonarakis

Correspondence

anton401@umn.edu

In brief

Tsai et al. identify that $CD56^+$ "NK-like" and $CD4^+CD8^+$ "double-positive" T cells were associated with a complete response to immunotherapy in a patient with high-grade MMR-deficient prostate cancer. These unique lymphocytes also expanded in other immunotherapy-responsive patients with diverse tumor types. These studies uncover possible conserved mechanisms of immunotherapy efficacy.

Tsai et al., 2026, Cell Reports Medicine 7, 102889
July 21, 2026 © 2026 The Author(s). Published by Elsevier Inc.
https://doi.org/10.1016/j.xcrm.2026.102889

CellPress

Cell Reports Medicine

CellPress
OPEN ACCESS

Report

Case of complete response to immunotherapy in MMR-deficient prostate cancer associated with NK-like and CD4$^+$CD8$^+$ T cells

Alexander K. Tsai,[1,2,3,14] John R. Lozada,[1,2,14] Philippa R. Kennedy,[1,2] David Moline,[1,2] Rachana Pandey,[4,5] Riley C. Lyons,[1,2] Christine Luo,[1,2] Rulin Wang,[6] Ali T. Arafa,[7] Elise L. Femino,[1,2] Sarah Zipkowitz,[6] Alexis Figueroa,[8] Patrick J. McCann,[9] Matthew C. Dallos,[9] Andrew Elliott,[10] Paari Murugan,[11] Martin Felices,[1,2] Nicholas A. Zorko,[1,2] Badrinath R. Konety,[12] Scott M. Dehm,[2,11] Jeffrey S. Miller,[1,2] Steven S. Shen,[4,5] Elizabeth A. Thompson,[13] Laura A. Sena,[6] Srinivasan Yegnasubramanian,[6] Justin Hwang,[1,2] and Emmanuel S. Antonarakis[1,2,15,*]

[1]Division of Hematology, Oncology and Transplantation, Department of Medicine, University of Minnesota, Minneapolis, MN 55455, USA
[2]Masonic Cancer Center, University of Minnesota, Minneapolis, MN 55455, USA
[3]Center for Immunology, University of Minnesota, Minneapolis, MN 55455, USA
[4]Institute for Health Informatics, University of Minnesota, Minneapolis, MN 55455, USA
[5]Clinical Translational Science Institute, University of Minnesota, Minneapolis, MN 55415, USA
[6]Sidney Kimmel Comprehensive Cancer Center, Johns Hopkins University School of Medicine, Baltimore, MD 21287, USA
[7]Department of Pharmacology, University of Minnesota, Minneapolis, MN 55455, USA
[8]Division of Infectious Diseases, Department of Medicine, Johns Hopkins University School of Medicine, Baltimore, MD 21205, USA
[9]Genitourinary Oncology Service, Department of Medicine, Memorial Sloan Kettering Cancer Center, New York, NY 10065, USA
[10]Caris Life Sciences, Phoenix, AZ 85040, USA
[11]Department of Laboratory Medicine and Pathology, University of Minnesota, Minneapolis, MN 55455, USA
[12]Allina Health Cancer Institute, Minneapolis, MN 55407, USA
[13]Department of Molecular Microbiology and Immunology, Bloomberg School of Public Health, Johns Hopkins University, Baltimore, MD 21205, USA
[14]These authors contributed equally
[15]Lead contact
*Correspondence: anton401@umn.edu
https://doi.org/10.1016/j.xcrm.2026.102889

SUMMARY

Mismatch repair deficiency (dMMR) and microsatellite instability (MSI-H) are rare in prostate cancer, occurring in 2%–4% of cases. These defects result in increased genomic instability and elevated tumor mutational burden (TMB), which can support responses to immune checkpoint inhibitors (ICIs). Here, we report a patient with locally advanced Gleason 5 + 5 = 10 prostatic adenocarcinoma harboring *MSH2* and *MSH6* genomic deletions with ultrahigh TMB (>250 mutations/megabase) in whom pembrolizumab resulted in a striking complete radiographic, pathologic, and molecular response. Using digital-spatial microscopy, single-cell RNA/T cell receptor (TCR) sequencing, and multiplex cytometry, we identify atypical tumor-infiltrating T cells with natural killer-like phenotypes and CD4$^+$CD8$^+$ (double-positive) lymphocytes. These clonal T cell populations expand preferentially following ICI and adopt terminally differentiated and cytotoxic profiles that may drive clinical response. Similar T cells are also present in diverse cancers and expand exclusively in ICI-responsive patients. These findings inform on the cellular mechanisms by which immunotherapies may mediate profound responses in patients with dMMR solid tumors.

INTRODUCTION

Although the prognosis of early-stage prostate cancer (PC) is generally favorable, high-grade prostatic malignancies are associated with significantly poorer outcomes.[1,2] Despite advances in local and systemic therapies, recurrences following curative-intent treatment are frequent in Gleason 5 + 5 = 10 PC.[1,2]

Immune checkpoint inhibitors (ICIs) have been investigated extensively for metastatic PC and are generally ineffective in molecularly unselected patients.[3] However, rare PC patients with mismatch repair deficiency (dMMR)/microsatellite instability (MSI-H) and/or high tumor mutational burden (TMB) occasionally respond favorably to ICI.[4,5] The mechanisms governing ICI sensitivity continue to evolve, and whether patients with dMMR/MSI-H malignancies exhibit unique immunological responses to ICI remains unknown.

Here, we report a remarkable case of a patient with locally advanced, Gleason 5 + 5 = 10, dMMR/MSI-H PC with ultrahigh TMB, who achieved a complete pathological and molecular response with pembrolizumab that was associated with clonal

© 2026 The Author(s). Published by Elsevier Inc.
This is an open access article under the CC BY license (http://creativecommons.org/licenses/by/4.0/).

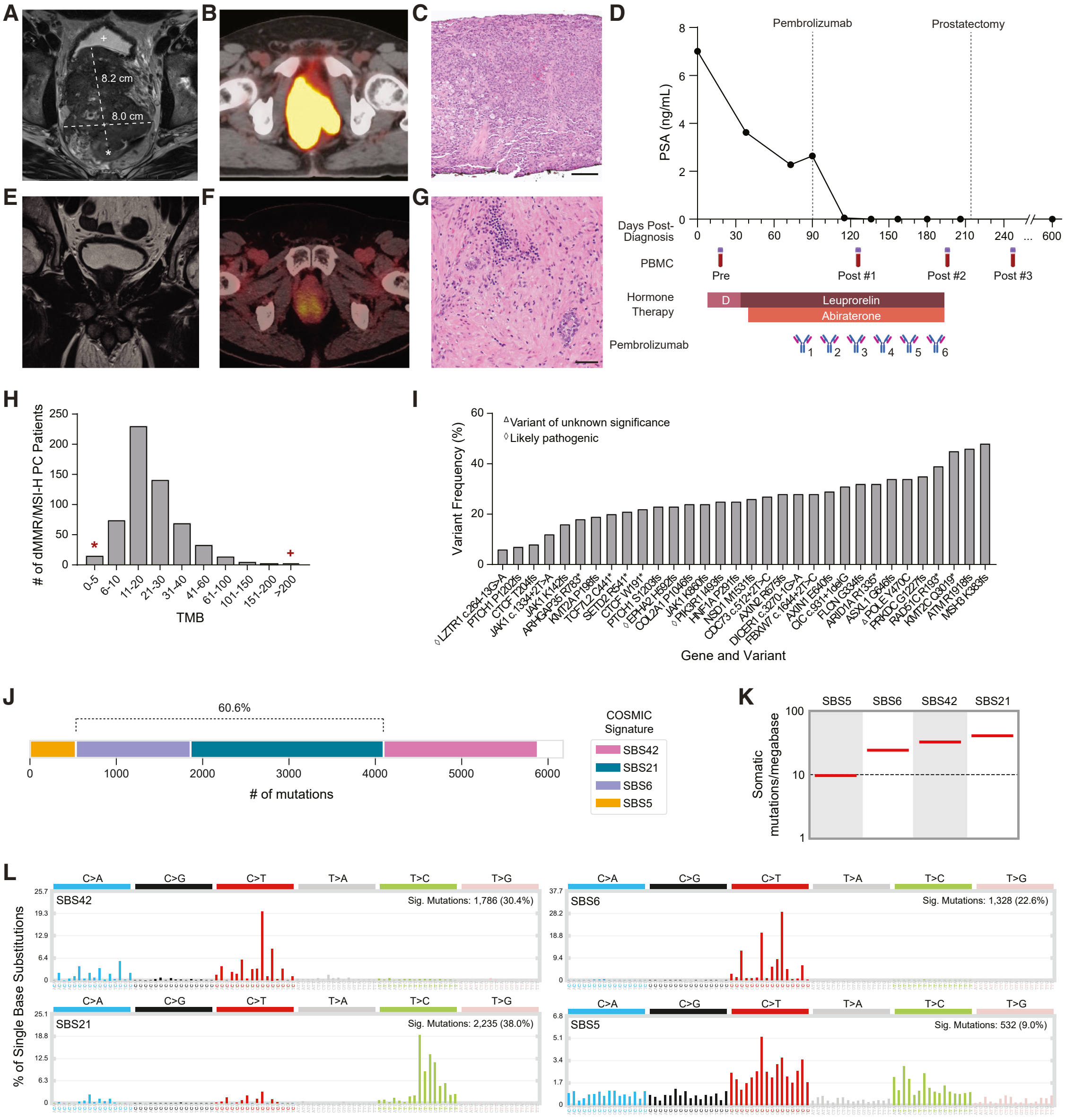

Figure 1. Clinical case history, genomic analysis, and histologic evaluation of tumor-infiltrating immune cells

(A) T2-weighted transverse view of prostatic MRI performed prior to treatment during diagnostic workup. Measurements of the large prostatic mass are shown along with rectal (*) and bladder (+) invasion.

(B) ^{18}F-PSMA-PET/CT imaging performed prior to treatment during diagnostic workup.

(C) Representative H&E section of Gleason 5 + 5 = 10 (Grade Group 5) prostatic acinar adenocarcinoma from a pre-treatment prostate biopsy. Scale bar, 100 μm.

(D) Serum prostate-specific antigen (PSA) measurements from the patient following diagnosis. Therapies administered are shown (D, degarelix). Serial peripheral blood mononuclear cell (PBMC) collections analyzed in downstream assays are also depicted.

(E) T2-weighted transverse view of prostatic MRI performed after four cycles of pembrolizumab.

(F) ^{68}Ga-PSMA-PET/CT imaging performed after four cycles of pembrolizumab.

(G) Representative H&E section of the post-ICI prostatectomy sample with chronic inflammation and fibrosis along with atrophic glands. Prostatic adenocarcinoma was not detected. Scale bar, 50 μm.

(H) Histogram of tumor mutational burden (TMB) from 584 dMMR (assessed by IHC) and/or MSI-H (assessed by DNA sequencing) prostate cancer patients from the Caris Life Sciences CODEai database. *, median TMB (3 mutations/megabase) from all PC patients (including pMMR/MSS patients) within this database. +, patient TMB (266 mutations/megabase).

(I) Pathogenic and likely pathogenic DNA alterations detected from whole-exome NGS analysis performed on diagnostic PC biopsies with variant allele frequencies (VAFs). Likely pathogenic variants and a variant of unknown significant (VUS) in *POLE* are indicated; all other variants were classified as pathogenic.

(legend continued on next page)

expansion of natural killer (NK)-like and double-positive (DP) $CD4^+CD8^+$ T cells. These NK-like and DP T cells expanded exclusively in ICI-responsive patients when examining larger PC and non-prostatic dMMR/MSI-H cohorts.

RESULTS

Clinical case history, genomic analysis, and histologic evaluation of tumor-infiltrating immune cells

A 67-year-old man presented with pelvic pain, hematuria, and hematochezia. MRI and prostate-specific membrane antigen (PSMA)-positron emission tomography (PET)/computed tomography (CT) imaging revealed an 8-cm prostatic mass with local invasion into the bladder and rectum, but without distant metastases (Figures 1A and 1B). Prostate biopsy demonstrated sheets of malignant cells with near-complete replacement of normal glands, indicative of Gleason 5 + 5 = 10 acinar adenocarcinoma, involving 100% of all cores (Figure 1C). This represented stage IIIC (cT4N0M0) disease.

Somatic whole-exome next-generation sequencing (NGS) of the tumor revealed dMMR/MSI-H disease harboring homozygous genomic deletions of *MSH2* and *MSH6* loci with accompanying protein loss. Germline genetic testing was unremarkable, without evidence for Lynch syndrome.

Androgen deprivation therapy (ADT) was initiated using degarelix 240 mg followed by leuprolide 45 mg and abiraterone 1,000 mg daily. While prostate-specific antigen (PSA) initially declined, the patient rapidly developed castration-resistant PC after only 3 months (Figure 1D). At that time, pembrolizumab (200 mg intravenously, in 21-day cycles) was administered for six cycles. This resulted in a brisk PSA decline to undetectable levels after two ICI cycles. Repeat MRI after four cycles of pembrolizumab revealed a normal-sized prostate gland measuring 3.3 $\times$ 3.2 $\times$ 2.4 cm with post-treatment fibrotic changes but without evidence of residual tumor (Figure 1E). No focal radiotracer avidity was detected on repeat PSMA-PET/CT imaging after four cycles of ICI, indicative of a complete radiographic response (Figure 1F).

Radical prostatectomy with pelvic lymphadenectomy was subsequently performed 1 month after the final pembrolizumab dose. Intraoperatively, significant fibrosis was noted between the prostate and rectum. Strikingly, histopathological examination revealed only glandular prostatic atrophy, fibrosis, and chronic inflammation without residual adenocarcinoma (Figure 1G). Five pelvic nodes were also negative for carcinoma. Repeat whole-exome NGS from the post-ICI prostatectomy specimen, which detects single-base substitutions (SBSs) derived from samples with 1%–2% tumor involvement, did not identify any somatic DNA alterations detected in the pre-treatment tumor biopsy. Thus, the patient was deemed to have a complete radiographic, pathologic, and molecular response. No additional adjuvant therapies were administered. The patient remains recurrence free, biochemically and radiographically, more than 18 months following prostatectomy with non-castrate testosterone levels.

Genomic, transcriptomic, cytometric, and spatial imaging approaches were used to serially profile immunological evolution in response to ICI. Whole-exome sequencing analysis (Table S1) revealed a TMB of 266 mutations/megabase—approximately 10-fold higher than the median TMB identified in a large cohort of 584 dMMR/MSI-H PCs (Figure 1H). In addition to *MSH2*/*MSH6* deletions, somatic pathogenic variants were present in several other genes involved in DNA damage/response including *ATM*, *KMT2C*, *MSH3*, *RAD51C*, *PRKDC*, and *SETD2* (Figure 1I), which may have contributed to the markedly elevated TMB. Mutational signature analysis was performed given that a variant of unknown significance (VUS) was also identified in DNA polymerase epsilon (*POLE*), which could have further influenced mutation accumulation. Catalog of Somatic Mutations in Cancer (COSMIC) SBS6 and SBS21 mutational signatures, associated with dMMR/MSI-H, accounted for 61% of the genomic alterations identified by NGS on pre-treatment biopsies (Figures 1J–1L). Polymerase deficiency mutational signatures were not detected, suggesting that the *POLE* VUS represented a passenger mutation.

Digital spatial microscopy with co-detection by indexing (CODEX) was performed on pre-treatment (tumor biopsy) and post-ICI (prostatectomy) specimens to interrogate microenvironment changes. Immune infiltration patterns varied distinctly in the two specimens: while diffuse infiltration characterized pre-treatment biopsies, immune cells in the prostatectomy specimen were confined to glands and were virtually absent in fibrotic zones that developed following ICI-induced tumor eradication (Figures S1A and S1B). Cycling $CD45^+$ leukocytes were observed exclusively within biopsy tissue, and ~40% of tumor cells were also actively proliferating (Figure S1C). PD-L1 was detected only in biopsies and expressed primarily on $CD11c^+$ myeloid cells (Figure S1D). Regarding lymphocytes, while tissue-infiltrating T cells were comparable in both samples, antigen-experienced CD45RO and PD-1-expressing $CD4^+$ and $CD8^+$ T cell subsets were enriched >2-fold in pre-treatment biopsies, as were proliferating Ki-67$^+$ T cells (Figures S1D and S1E), suggesting the development of tumor-specific T cells that contracted following therapy. Additionally, atypical T cell phenotypes were identified in tumor tissues. Specifically, CD56, a marker of NK and NKT cells, was detected on "NK-like" $CD8^+$ T cells (Figure S1F). Unconventional co-expression of $CD4^+$ and $CD8^+$ was also observed in a subset of DP T cells (Figure S1G). These unique T cells expressed PD-1 in pre-treatment biopsies, suggestive of tumor specificity.

(J) Number of mutations matching the four Catalog of Somatic Mutations in Cancer (COSMIC) single-base-substitution (SBS) mutational signatures identified: SBS5, SBS6, SBS21, and SBS42. SBS6 and SBS21 are associated with dMMR/MSI-H defects. SBS5 has unknown etiology, whereas SBS42 is associated with exposure to haloalkanes.

(K) Number of somatic mutations per megabase based on COSMIC SBS mutational signature.

(L) Characteristic substitutions associated with each signature, and their prevalence among all variants. No variants were associated with DNA polymerase deficiency COSMIC signatures.

See also Figure S1 and Table S1.

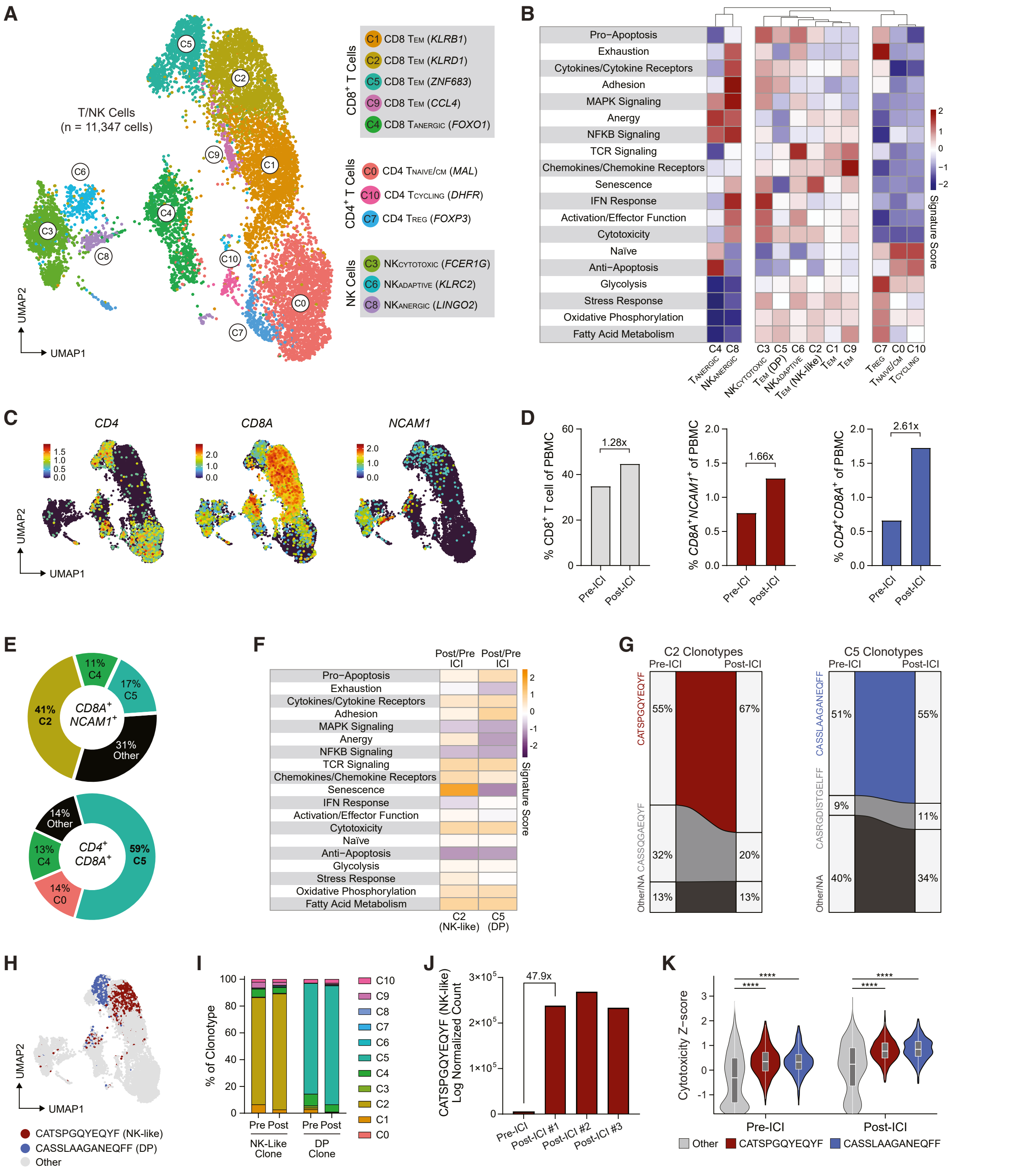

Figure 2. Clonal NK-like and CD4+CD8+ double-positive T cells adopted signatures of effector functionality

(A) Single-cell RNA sequencing showing hierarchical NK cell and T cell clusters by uniform manifold approximation and projection (UMAP) from pre-ICI and post-ICI time point #1 (see Figure 1D). Cell clusters were annotated as CD8 effector memory T cells (T_{EM}; C1, C2, C5, and C9), CD8 anergic T cells (C4), naive/central memory CD4 T cells ($T_{NAIVE/CM}$; C0), cycling CD4 T cells ($T_{CYCLING}$; C10), regulatory CD4 T cells (T_{REG}; C7), cytotoxic NK cells ($NK_{CYTOTOXIC}$; C3), adaptive NK cells ($NK_{ADAPTIVE}$; C6), and anergic NK cells ($NK_{ANERGIC}$; C8). Defining genes for each cluster are listed.

(B) T cell function gene signature scores (see Chu et al.[6]) for NK cell and T cell clusters (pre- and post-ICI time points are combined).

(C) *CD4* (left), *CD8A* (middle), and *NCAM1* (right) gene expression (pre- and post-ICI time points are combined).

(D) Frequencies and fold change of CD8+ T cells (left), *CD8A*+*NCAM1*+ NK-like T cells (middle), and *CD4*+*CD8A*+ DP T cells (right) of total PBMCs at pre-ICI and post-ICI time points.

(E) T cell clusters represented among *CD8A*+*NCAM1*+ NK-like T cells (upper) or *CD4*+*CD8A*+ DP T cells (lower).

(legend continued on next page)

Clonal NK-like and $CD4^+CD8^+$ double-positive T cells adopted signatures of effector functionality

Single-cell RNA sequencing (scRNA-seq) was performed on peripheral blood mononuclear cells (PBMCs) from pre-treatment (PBMC Pre) and initial post-ICI (PBMC Post #1) time points (Figures 1D, S2A, and S2B), given an expectation that pivotal immunological changes would occur shortly following ICI initiation. NK cells and T cells segregated into 11 clusters and were interrogated more deeply due to their frequent association with ICI response (Figure 2A). Most $CD8^+$ T cell clusters (C1, C2, C5, and C9) resembled effector memory (T_{EM}) subsets that curiously expressed various NK-associated genes (Figures 2A and S2C). Signatures of T cell function[6] were similar between $CD8^+$ T_{EM} and non-anergic NK cell clusters (Figure 2B), reinforcing that T_{EM} populations adopted gene expression patterns similar to NK cells. Aligning with CODEX findings, *NCAM1*$^+$ (encoding CD56) NK like T cells and *CD4*$^+$*CD8A*$^+$ co-expressing DP T cells were identified (Figure 2C). These unique T cell subsets exhibited preferential expansion following ICI treatment compared with the overall $CD8^+$ T cell population (Figure 2D). The NK-like and DP T cells predominantly populated clusters C2 and C5, respectively (Figure 2E). Additionally, signatures of T cell function, including glycolysis, chemokine receptor signaling, T cell receptor (TCR) signaling, effector function, and cytotoxicity, were augmented in the NK-like (C2) and DP (C5) T cells following ICI administration (Figure 2F). Overall, effector memory NK-like and DP T cells expanded after ICI, suggesting that these T cell subsets might contribute to anti-tumor immunity.

Single-cell TCR sequencing was next used to evaluate T cell clones within NK-like and DP T cells. Notably, Vα24/Vβ18- and Vα7.2-based TCR chains were not detected, confirming that C2 and C5 cells represented αβ T cells as opposed to NKT or mucosal-associated innate T cells. Strikingly, two unique TCR clones comprised >50% of T cells within clusters C2 and C5 (Figure 2G). These two TCR clones, CATSPGQYEQYF in C2 and CASSLAAGANEQFF in C5, expanded following ICI and remained confined to their respective clusters (Figures 2H and 2I), suggesting terminal differentiation. The dominant NK-like and DP clones were comparatively rare in clusters other than C2 and C5 (Figure S3A). By orthogonal bulk TCR sequencing, both clones were among only eight TCRs that were shared between pre-treatment tumor biopsies and circulating T cells (Figure S3B), indicating that NK-like and DP T cells infiltrated the patient's tumor. DP T cells expressing the CASSLAAGANEQFF TCR formed the tenth most prevalent intratumoral T cell clone, whereas NK-like T cells harboring the CATSPGQYEQYF TCR were the second most dominant intratumoral clone (Figure S3C). Notably, the CATSPGQYEQYF clone mapping to NK-like T cells markedly expanded in circulation by 47.9-fold after ICI (Figure 3J), emerging as the second most abundant circulating TCR clone at all three post-ICI time points (Figure S3B). Thus, ICI was associated with a dramatic and durable expansion of NK-like T cells.

Overall clonal diversity within circulating T cells contracted following ICI initiation, consistent with evolving dominance of select tumor-specific clones (Figure S3D). The most prevalent NK-like and DP T cell clones also exhibited increased signatures of cytotoxicity compared with all other $CD8^+$ T cell clones (Figure 2K), further supporting possible tumor specificity. Altogether, clonal NK-like and DP T cells expanded in circulation following ICI and adopted effector-associated functions that were further enhanced by ICI. As these atypical cells were also detected in pre-treatment tumor tissue and exhibit anti-tumor potential,[7–9] they likely represent relevant tumor-specific populations.

Terminally differentiated NK-like and $CD4^+CD8^+$ double-positive T cells exhibited cytotoxic potential

NK-like and DP T cells at all four PBMC time points (Figure 3A) were further evaluated using orthogonal multiparameter spectral flow cytometry and cytometry by time of flight (CyTOF), and compared with healthy controls (HCs). Patient and age- and sex-matched HC PBMCs diverged by principal component analysis (Figure 3B), and NK-like and DP T cells segregated using unbiased dimensional analysis (Figure 3C). Two distinct DP populations were identified—$CD4^{Hi}CD8^{Lo}$ and $CD4^{Lo}CD8^{Hi}$—aligning with foundational descriptions of DP T cells.[10] $CD4^{Hi}CD8^{Lo}$ DP cells, along with NK-like $CD56^+CD4^+$ and $CD56^+CD8^+$ T cell populations were 3- to 9-fold more prevalent within the T cell compartment of the patient compared with HCs (Figures 3D and S4A), suggesting that these populations expanded in response to PC development.

Canonical T cell subsets including naive, central memory (T_{CM}), T_{EM}, and terminally differentiated $CD45RA^+$ T_{EMRA} were examined within both $CD4^+$ and $CD8^+$ T cells. A marked increase in T_{EMRA} frequency was observed within both the $CD4^+$ (>10-fold) and $CD8^+$ (∼2-fold) compartments compared with HCs (Figures 3E, 3F, S4B, and S4C). CD56 was expressed predominantly within T_{EMRA}, and these cells were increased 2- to 5-fold compared with HC lymphocytes (Figures 3G–3J and S4D–S4G). Patient T_{EMRA} cells were also enriched for cytotoxic perforin and granzyme B compared with all other T cell subsets (Figures 3K, 3L, S4H, and S4I). Thus, NK-like T cells formed terminally differentiated effector-like cells in circulation.

Regarding DP T cells, although two populations were identified, only $CD4^{Hi}CD8^{Lo}$ T cells expanded following ICI treatment

(F) Fold change in T cell signature scores within NK-like T cell cluster C2 and DP T cell cluster C5 between post-ICI and pre-ICI time points.
(G) T cell receptor (TCR) clonotypes from single-cell TCR sequencing represented among cluster C2 (left) and cluster C5 (right) at pre-ICI and post-ICI time points. The designation "Other/NA" includes all other TCR clonotypes and cells lacking TCR data.
(H and I) Distribution of CATSPGQYEQYF (NK-like T cells), CASSLAAGANEQFF (DP T cells), and other TCR clones among NK/T cell clusters (H) with quantifications at pre-ICI and post-ICI time points (I).
(J) CATSPGQYEQYF (NK-like T cell) clonotype count at all four time points by bulk TCR sequencing.
(K) Cytotoxicity signature scores for CATSPGQYEQYF (NK-like) and CASSLAAGANEQFF (DP) clones compared with all other clones at pre-ICI and post-ICI time points. Wilcoxon tests were used for comparisons and statistical significance denoted as ****$p < 0.0001$.
See also Figures S1–S3.

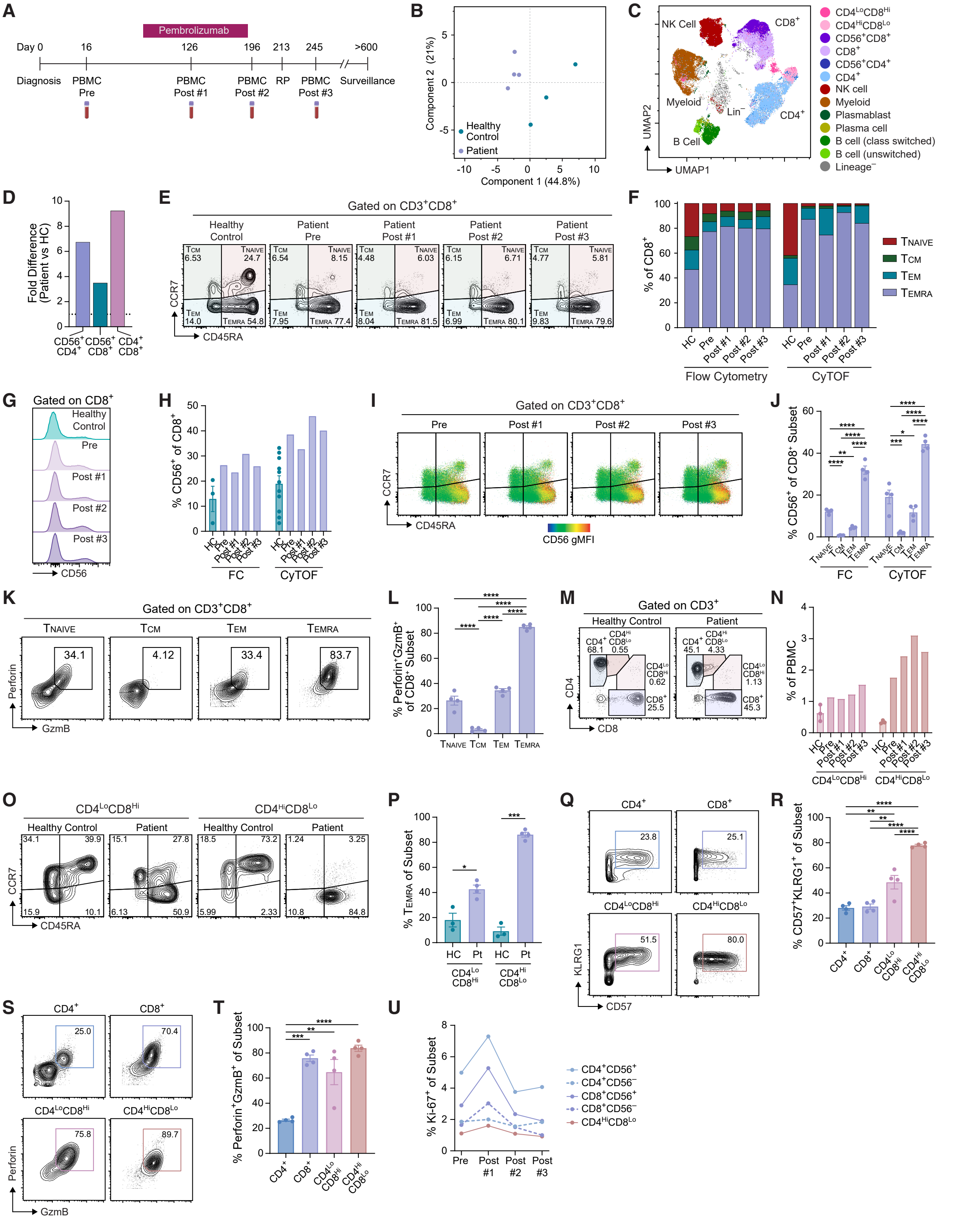

(legend on next page)

(Figures 3M and 3N). Similar to NK-like T cells, CD4^{Hi}CD8^{Lo} DP T cells exclusively adopted T$_{EMRA}$ phenotypes, which differed distinctly from differentiation patterns adopted by rare CD4^{Hi}CD8^{Lo} cells in HCs (Figures 3O and 3P). Additionally, patient-derived CD4^{Hi}CD8^{Lo} DP T cells lacked memory-associated markers (CD27, CD127; Figures S4J and S4K), acquired markers associated with effector T cells and NK cells (CD56, CD57, CD161, and KLRG1; Figures 3Q, 3R, S4L, and S4M), and produced robust levels of cytotoxic perforin and granzyme B (Figures 3S and 3T). While NK-like and DP T cells adopted phenotypes consistent with terminally differentiated T$_{EMRA}$, PD-1 was not detected (Figure S5A), suggestive of lack of recent antigen exposure and making functional exhaustion of these circulating cells unlikely. Notably, Ki-67$^+$ cycling CD4$^+$CD56$^+$, CD8$^+$CD56$^+$, and CD4^{Hi}CD8^{Lo} cells increased following ICI, and CD56$^+$ T cells exhibited greater proliferation relative to their CD56$^-$ counterparts (Figure 3U). Collectively, clonal CD56$^+$ NK-like and CD4$^+$CD8$^+$ DP T cells were more prevalent at baseline compared with HCs, infiltrated tumors, expanded/proliferated after ICI, and adopted terminally differentiated phenotypes poised to exert cytotoxic activity.

Cytotoxic NK-like and CD4$^+$CD8$^+$ DP T cells expand in cancer patients responding to immunotherapy

To extend our findings, we evaluated additional cancer patient cohorts treated with ICI. To the best of our knowledge, PC cohorts with dMMR/MSI-H defects do not exist due to the rarity of these defects in PC. While ICI use is also rare in PC, a recent clinical trial of patients treated with neoadjuvant Fc-enhanced anti-CTLA4 (aCTLA4) immunotherapy by Ager et al.[11] allowed for evaluation of PC patients treated with ICI. CD8$^+$ T cells from CyTOF performed on intratumoral samples from patients treated with combination ADT plus aCTLA4 were analyzed by uniform manifold approximation and projection and compared with untreated patients. We first reaffirmed previously reported findings that a population of tumor-infiltrating 41BB$^+$CD39$^+$ CD8$^+$ T cells exclusively formed in patients treated with aCTLA4 (Figures 4A–4C).[11] Most of these cells also co-expressed CD56 and resembled our previously characterized NK-like T cells. Tumor-infiltrating CD56$^+$CD8$^+$ T cells arising uniquely in aCTLA4-treated patients also expressed PD-1, reinforcing possible tumor reactivity. While total CD8$^+$ T cells were comparable in untreated and aCTLA4-treated patients (Figure 4D), 41BB$^+$CD39$^+$CD56$^+$ CD8$^+$ T cell frequencies were significantly enriched in patients treated with aCTLA4 and nearly undetectable in untreated patients (Figure 4E). A CD4$^+$CD8$^+$ DP T cell population that similarly expressed 41BB, CD39, and CD56 was also significantly enriched in aCTLA4-treated patients (Figures 4F–4H). As 41BB is induced in activated T cells[12] and CD39 expression is linked to tumor-antigen specificity,[13] ICIs may promote development of activated tumor-specific NK-like and DP T cells in PC.

To further evaluate if NK-like and/or DP T cells were present in patients with dMMR/MSI-H cancers, we analyzed scRNA-seq from Chen et al.,[14] in which tumor-infiltrating T cells were tracked in 12 patients with dMMR/MSI-H colorectal cancer (CRC) that responded to neoadjuvant treatment with anti-PD-1 ICIs.

Figure 3. Terminally differentiated NK-like and CD4$^+$CD8$^+$ double-positive T cells exhibited cytotoxic potential

(A) Schematic of PBMC collections in relation to pembrolizumab treatment. PBMCs from all four time points were interrogated using 29-parameter spectral flow cytometry and 42-parameter cytometry by time-of-flight (CyTOF) analyses. PBMCs from healthy donors (3 for flow cytometry [age and sex matched] and 11 for CyTOF) served as healthy control (HC) samples.

(B) Principal component analysis comparing patient PBMCs at each time point to HC PBMCs using spectral flow cytometry.

(C) UMAP dimensional analysis of concatenated patient PBMCs from all four time points assessed by spectral flow cytometry, with manual annotation of the indicated cell populations.

(D) Fold difference in frequencies of the indicated populations within the T cell compartment when comparing patient T cell subsets (averaged between all four time points) with HC T cell subsets (averaged between the three healthy controls) assessed using spectral flow cytometry.

(E and F) Representative plots (E) of frequencies of T$_{NAIVE}$ (CCR7$^+$CD45RA$^+$), T$_{CM}$ (CCR7$^+$CD45RA$^-$), T$_{EM}$ (CCR7$^-$CD45RA$^-$), and T$_{EMRA}$ (CCR7$^-$CD45RA$^+$) within the CD8$^+$ T cell compartment of patient and HC PBMCs (F) assessed by spectral flow cytometry and CyTOF.

(G) Histograms of CD56 on CD8$^+$ T cells from spectral flow cytometry showing a representative HC and the patient at all four time points.

(H) CD56$^+$ frequencies of CD8$^+$ T cells from patient and HC PBMCs assessed using flow cytometry and CyTOF.

(I and J) Heatmaps of CD56 geometric mean fluorescent intensity (gMFI; I) with CD56$^+$ frequencies based on T$_{NAIVE}$, T$_{CM}$, T$_{EM}$, and T$_{EMRA}$ CD8$^+$ T cell subsets (combined across all four time points; J) assessed using spectral flow cytometry and CyTOF. ANOVA with Tukey tests were used for comparisons with statistical significance denoted as *$p < 0.05$, **$p < 0.01$, ***$p < 0.001$, and ****$p < 0.0001$.

(K and L) Representative plots (K) of perforin$^+$granzyme B$^+$ (GzmB$^+$) frequencies of T$_{NAIVE}$, T$_{CM}$, T$_{EM}$, and T$_{EMRA}$ CD8$^+$ T cell subsets combined across all four time points (L) assessed using CyTOF. ANOVA with Tukey tests were used for comparisons, with statistical significance denoted as ****$p < 0.0001$.

(M and N) Representative plots (gated on CD45$^+$CD3$^+$ T cells; M) of frequencies of CD4$^+$, CD8$^+$, CD4^{Lo}CD8^{Hi}, and CD4^{Hi}CD8^{Lo} T cells within patient and HC PBMCs (N) assessed using spectral flow cytometry.

(O and P) Representative plots (O) of frequencies of T$_{EMRA}$ within either CD4^{Lo}CD8^{Hi} or CD4^{Hi}CD8^{Lo} T cell subsets of patient (combined across all four time points) and HC PBMCs (P) assessed using spectral flow cytometry. Unpaired *t* tests were used for comparisons with statistical significance denoted as *$p < 0.05$ and ***$p < 0.001$.

(Q and R) Representative plots (Q) of frequencies of effector T cell-associated CD57$^+$KLRG1$^+$ cells within CD4$^+$, CD8$^+$, CD4^{Lo}CD8^{Hi}, and CD4^{Hi}CD8^{Lo} patient-derived T cells combined across all four time points (R) assessed using CyTOF. ANOVA with Tukey tests were used for comparisons with statistical significance denoted as **$p < 0.01$ and ****$p < 0.0001$.

(S and T) Representative plots (S) of frequencies of cytotoxicity-associated perforin$^+$granzyme B$^+$ cells within CD4$^+$, CD8$^+$, CD4^{Lo}CD8^{Hi}, and CD4^{Hi}CD8^{Lo} patient-derived T cells combined across all four time points (T) assessed using CyTOF. ANOVA with Tukey tests were used for comparisons with statistical significance denoted as **$p < 0.01$, ***$p < 0.001$, and ****$p < 0.0001$.

(U) Frequencies of proliferating Ki-67$^+$ cells within CD56$^-$CD4$^+$, CD56$^+$CD4$^+$, CD56$^-$CD8$^+$, CD56$^+$CD8$^+$, and CD4^{Hi}CD8^{Lo} T cell subsets across all four time points assessed using CyTOF.

All panels show mean ± SEM. See also Figures S4 and S5.

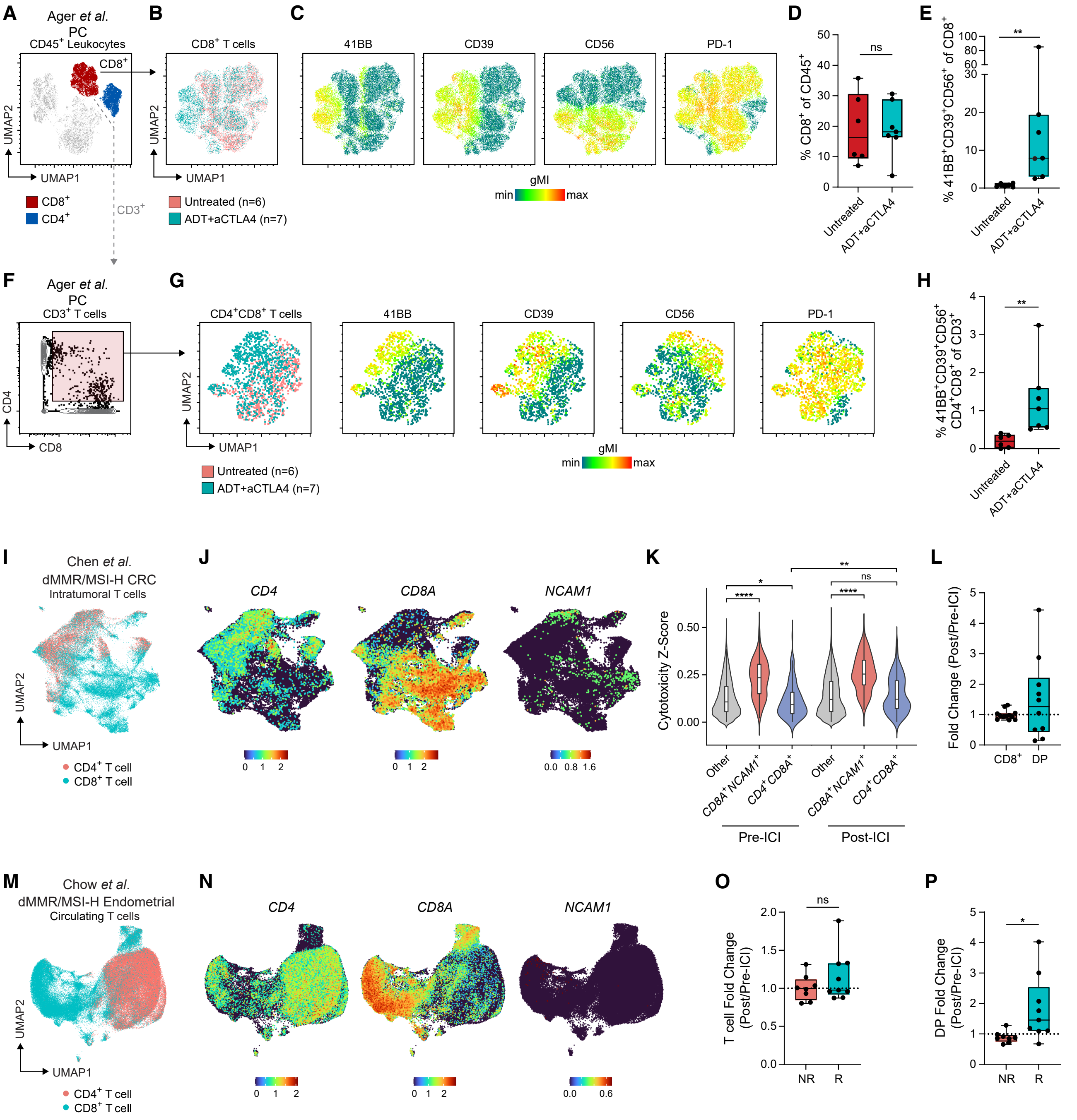

Figure 4. Cytotoxic NK-like and CD4$^+$CD8$^+$ DP T cells expand in cancer patients responding to immunotherapy

(A) UMAP analysis of concatenated intratumoral CD45$^+$ leukocytes from CyTOF performed on PC patients treated with or without neoadjuvant ADT plus aCTLA4 (see Ager et al.[11]) with CD4$^+$ and CD8$^+$ T cells annotated.

(B) UMAP analysis of concatenated CD8$^+$ T cells, with cells from each treatment group annotated.

(C) Heatmaps of concatenated CD8$^+$ T cells depicting geometric mean intensity (gMI) of 41BB, CD39, CD56, and PD-1.

(D) CD8$^+$ T cell frequency of CD45$^+$ leukocytes. ns, not significant by Mann-Whitney test.

(E) 41BB$^+$CD39$^+$CD56$^+$ frequency of CD8$^+$ T cells. Mann-Whitney test was used for comparison with statistical significance denoted as $^{**}p < 0.01$.

(F) Concatenated CD3$^+$ T cells with CD4$^+$CD8$^+$ DP gated.

(G) UMAP analysis of concatenated CD4$^+$CD8$^+$ DP T cells and gMI heatmaps of 41BB, CD39, CD56, and PD-1.

(H) 41BB$^+$CD39$^+$CD56$^+$CD4$^+$CD8$^+$ frequency of CD3$^+$ T cells. Mann-Whitney test was used for comparison with statistical significance denoted as $^{**}p < 0.01$.

(I) UMAP analysis of T cells from scRNA-seq of dMMR/MSI-H CRC patients treated with anti-PD-1 ICI (see Chen et al.[14]) with CD4$^+$ and CD8$^+$ T cells annotated.

(J) *CD4*, *CD8A*, and *NCAM1* gene expression in T cells from CRC patients.

(legend continued on next page)

CellPress
OPEN ACCESS

CD8A$^+$*NCAM1*$^+$ NK-like T cells exhibited significantly increased cytotoxicity signature scores at both pre-ICI and post-ICI time points compared with other T cells (Figures 4I–4K). While *CD4*$^+$*CD8A*$^+$ DP T cell cytotoxicity signatures were similar to those of other T cells, scores significantly increased following ICI. Moreover, DP T cells expanded at least to some degree in 60% of patients, whereas overall CD8$^+$ T cell populations were largely unchanged after ICI treatment (Figure 4L). Therefore, NK-like T cells may exhibit increased anti-tumor activity compared with other T cell subsets in CRC patients with dMMR/MSI-H tumors, and DP T cells selectively expand following ICI.

Most patients with dMMR/MSI-H CRC respond favorably to ICI, restricting our ability to perform comparisons between responders and nonresponders. Consequently, we performed similar evaluations of circulating T cells in dMMR/MSI-H endometrial carcinoma patients by scRNA-seq from Chow et al.,[15] where ICI responses were more variable. While *CD4*$^+$*CD8A*$^+$ DP T cells were identified, NK-like T cells were rarely detected (Figures 4M and 4N), potentially due to differences in cancer type, intratumoral versus circulating T cell frequencies, or technical limitations in sequencing. Although total circulating T cell frequencies were largely unchanged after treatment in both ICI responders and nonresponders, DP T cells exclusively expanded in responders (Figures 4O and 4P). To expand our findings to MMR-proficient (pMMR) cancers, we evaluated tumor-infiltrating *CD4*$^+$*CD8A*$^+$ DP T cells from triple-negative breast[16] and head/neck squamous cell carcinoma[17] patients treated with ICIs. Similar to findings in dMMR/MSI-H cancers, DP T cells expanded only in ICI responders (Figures S6A–S6F). Accordingly, DP expansion may correlate with ICI response in diverse pMMR and dMMR cancers.

DISCUSSION

Although T cells targeting neoantigens generated by hypermutated malignancies are foundational for responses to ICI, how these cells acquire anti-tumor potential remains unclear. Herein, we describe a patient with dMMR/MSI-H, ultra-mutated PC in whom pembrolizumab promoted clonal expansion of NK-like and CD4$^+$CD8$^+$ DP T cells. These unique cells may have been essential in rendering a durable, complete pathological and molecular response to ICI in a patient with Gleason 10 locally advanced PC—a striking clinical course not previously described.[18]

T cells acquiring NK-associated phenotypes are implicated in numerous diseases.[7,8] These unique lymphocytes can elicit potent cytotoxic activity against tumor cells, often using MHC-independent innate-like mechanisms.[7,8] Although NK-like T cells appear protective in certain hematologic malignancies,[19–23] inverse associations have been observed in several solid tumors.[24–28] In our patient, NK-like T cells were highly enriched in circulation compared with PBMCs from HCs and infiltrated tumor tissues. Additionally, the cells acquired terminally differentiated phenotypes and produced high levels of effector molecules—features consistent with established hallmarks of NK-like T cells.[7,8] Remarkably, a single NK-like T cell clone with cytotoxic potential expanded nearly 50-fold following initiation of ICI, supporting a possible role in complete eradication of this high-grade, castration-resistant PC. NK-like cells exhibiting phenotypes associated with activation and tumor specificity were also independently identified in patients treated with aCTLA4 ICI, reinforcing that immunotherapy may drive formation of NK-like T cells in PC. These cells may also be conserved in dMMR/MSI-H cancers, as they adopted cytotoxic profiles in CRC patients with such defects.

Regarding DP T cells, emerging evidence supports that they represent a mature lymphocyte population capable of MHC-I and/or MHC-II-restricted tumor-specific potential.[9] Akin to NK-like T cells, DP T cells have been reported to possess pro- or anti-tumor functions and frequently express markers associated with effector cytotoxic functions.[9] The origins of these cells have recently become clearer, with some groups hypothesizing that tumor-reactive DP T cells arise from CD4 or CD8 single-positive precursors and acquire shared functions of each canonical T cell type.[29–32] In the case herein, similar to NK-like T cells, CD4^{Hi}CD8^{Lo} DP T cells infiltrated the patient's tumor, were terminally differentiated, and displayed cytotoxic potential. Yet, as these cells were present prior to ICI treatment and only moderately increased after treatment, it remains unclear whether they exerted significant anti-tumor effects. Nevertheless, DP T cells appear to selectively expand in ICI responders across diverse cancers and, therefore, may represent a potential biomarker of ICI response.

In conclusion, our findings support NK-like and DP T cells as promising components of anti-tumor immunity. Additional investigations on the ontogeny of these cells and their potential to serve as immunotherapy targets, including approaches with purified CD56$^+$ and CD4$^+$CD8$^+$ T cells with tumor-specific receptors, are warranted. This case further reinforces the remarkable potential of ICIs in dMMR/MSI-H cancers with ultrahigh TMB, even in tumors not typically considered responsive to immunotherapies, and suggests that some MMR-deficient malignancies may be cured with nonoperative management.[18,33]

Limitations of the study

Although NK-like and DP T cells in our patient adopted several attributes suggestive of tumor specificity, attempts to infer

(K) Cytotoxicity signature scores for NK-like and *CD4*$^+$*CD8A*$^+$ cells compared with all other T cells at pre-ICI and post-ICI time points in CRC patients. Wilcoxon tests were used for comparisons with statistical significance denoted as *$p < 0.05$, **$p < 0.01$, and ****$p < 0.0001$. ns, not significant.
(L) Fold change in CD8$^+$ T cells and *CD4*$^+$*CD8A*$^+$ DP T cells following ICI treatment in CRC patients.
(M) UMAP analysis of T cells from scRNA-seq of dMMR/MSI-H endometrial carcinoma patients treated with pembrolizumab (see Chow et al.[15]).
(N) *CD4*, *CD8A*, and *NCAM1* gene expression in T cells from endometrial carcinoma patients.
(O) Fold change in T cells following pembrolizumab treatment in endometrial carcinoma patients segregated by nonresponders (NR) and responders (R). ns, not significant by Mann-Whitney test.
(P) Fold change in *CD4*$^+$*CD8A*$^+$ DP T cells following pembrolizumab treatment in endometrial carcinoma patients segregated by NR and R. Mann-Whitney test was used for comparison with statistical significance denoted as *$p < 0.05$.
Dots in box (median and interquartile range) and whisker (minimum and maximum) plots in (D, E, H, L, O, and P) represent individual patients. See also Figure S6.

TCR specificity were hampered by the patient's ultrahigh TMB and thousands of potential neoantigens. Emerging improvements in predictive *in silico* technologies may aid in identifying T cell specificity in similarly challenging situations. Advances in spatial and single-cell technologies will also continue to allow for validation of our unique findings while clarifying the contexts under which NK-like and DP T cells contribute to anti-tumor immunity. Our findings reflect a single-patient case study, albeit with deep molecular and immunologic characterization, and may not be generalizable to all dMMR/MSI-H and/or ultramutated cancers. To partially alleviate this limitation, we expanded our findings in prostate, dMMR/MSI-H colorectal, endometrial, breast, and head/neck cancer patient cohorts treated with ICIs. While several concordant trends were observed, these cohorts were too small to rigorously control for confounding variables that might influence NK-like and DP T cells. Additional larger and prospective studies are necessary to more accurately quantify associations between these cells and patient outcomes.

RESOURCE AVAILABILITY

Lead contact

Requests for further information and resources should be directed to and will be fulfilled by the lead contact, Emmanuel S. Antonarakis (anton401@umn.edu).

Materials availability

This study did not generate new unique reagents.

Data and code availability

- Local law prohibits depositing raw genomic datasets derived from human samples. Single-cell RNA-seq data have been deposited at GEO: GSE332846 and are publicly available as of the date of publication. This paper analyzes existing, publicly available data, accessible at DOIs: https://doi.org/10.1016/j.xcrm.2026.102638, https://doi.org/10.1016/j.ccell.2024.06.009, https://doi.org/10.1158/2159-8290.CD-22-0686, https://doi.org/10.1016/j.ccell.2021.09.010, and https://doi.org/10.1016/j.cell.2022.06.018.
- This paper does not report original code.
- Any additional information required to reanalyze the data reported in this paper is available from the lead contact upon request.

ACKNOWLEDGMENTS

We thank Kellie N. Smith, PhD, and Li Zhang at Johns Hopkins University for conducting bulk TCR sequencing. We thank the Johns Hopkins Experimental and Computational Genomics Core, supported by NCI P30CA006973, for assistance with the single-cell RNA and coupled TCR sequencing. We also thank Megan Larson and Rose Wangen at the University of Minnesota for performing CyTOF analysis. For multiplex imaging, we thank Liam McLaughlin, Thomas Pengo, and Myat Mo at the University of Minnesota, the Clinical and Translational Science Institute's Histology Core (University of Minnesota), and the Immune Monitoring Core at Mayo Clinic (Rochester, Minnesota) for analytical support. This work was supported by an NIH General Medical Sciences T32GM008244 grant (to J.R.L.); NCI grants F30CA294723 (to J.R.L.), R35CA283892 (to J.S.M. and M.F.), R37CA288972 (to J.H.), U54CA274370 (to S.Y.), P50CA272391 (to S.Y.), P01CA065493 (to P.R.K.), P01CA111412 (to P.R.K.), and P30CA077598 (to E.S.A.); DOD grant W81XWH-22-2-0025 (to E.S.A.); the Commonwealth and V Foundations (to S.Y.); and the Conquer Cancer Foundation (to J.R.L.).

AUTHOR CONTRIBUTIONS

Conceptualization, A.K.T., J.R.L., J.H., and E.S.A.; methodology, A.K.T., J.R.L., P.R.K., D.M., R.P., C.L., M.F., S.S.S., E.A.T., L.A.S., S.Y., J.H., and E.S.A.; investigation, A.K.T., J.R.L., P.R.K., D.M., R.P., R.C.L., C.L., R.W., E.L.F., S.Z., A.F., P.J.M., M.C.D., A.E., P.M., M.F., B.R.K., S.S.S., E.A.T., L.A.S., S.Y., and J.H.; writing – original draft, A.K.T.; writing – review and editing, J.R.L., P.R.K., D.M., R.P., A.T.A., A.E., P.M., N.A.Z., B.R.K., S.M.D., J.S.M., S.S.S., L.A.S., S.Y., J.H., and E.S.A.; funding acquisition, P.R.K., S.Y., J.H., and E.S.A.; resources, P.R.K., M.F., S.S.S., L.A.S., S.Y., J.H., and E.S.A.; supervision, J.H. and E.S.A.

DECLARATION OF INTERESTS

D.M. consults for Tempus. M.C.D. receives research support from Johnson & Johnson and Bristol Myers Squibb and personal fees from DAVA Oncology, Curio, Bayer, Bristol Myers Squibb, Astra Zeneca, Xilio, and Johnson & Johnson. J.S.M. has been a paid consultant for Fate Therapeutics and has received research funds and stock options from this relationship. J.S.M. and M.F. receive research support and, with the University of Minnesota, are shared owners of the TriKE technology licensed by the University to GT Biopharma, Inc. J.S.M. and M.F. also consult for and hold stock options in GT Biopharma, Inc. Fate and GT Biopharma may commercially benefit from the results of this research project. J.S.M. serves on the Scientific Advisory Board of Sanofi, Vycellix, and Simcha. N.A.Z. has received honoraria from the Association of Community Cancer Centers (ACCC) and Department of Defense Prostate Cancer Research Program, Mosaic Research Management, Slingshot Insights, and Bayer (Institutional); research funding to his institution from Janssen Research & Development, Johnson & Johnson, Janux Therapeutics, Amgen, Lava Therapeutics, GT Biopharma, ArsenalBio, and Takeda; and expenses from Telix Pharmaceuticals, Caris Life Sciences, Bayer, Janux Therapeutics, and DAVA Oncology and serves in an advisory capacity for Bayer and Takeda. L.A.S. has received funding to her institution from Panbela Therapeutics. J.H. consults for Tempus and Astrin Biosciences and is co-founder of EMRGNSE, LLC. E.S.A. receives grants and personal fees from Janssen, Johnson & Johnson, Sanofi, Bayer, Bristol Myers Squibb, Convergent Therapeutics, Curium, MacroGenics, Merck, Pfizer, and AstraZeneca; personal fees from Aadi Bioscience, Abeona Therapeutics, Aikido Pharma, Astellas, Amgen, Blue Earth, Boundless Bio, Corcept Therapeutics, Duality Bio, Exact Sciences, Hookipa Pharma, Invitae, Eli Lilly, Foundation Medicine, Menarini-Silicon Biosystems, Tango Therapeutics, Tempus, Tolmar Scientific, VIR Biotechnology, and Z-alpha; and grants from Novartis, Celgene, and Orion and has a patent for an AR-V7 biomarker technology that has been licensed to Qiagen.

DECLARATION OF GENERATIVE AI AND AI-ASSISTED TECHNOLOGIES IN THE WRITING PROCESS

During the preparation of this work, the authors did not use any generative AI or AI-assisted technologies.

STAR★METHODS

Detailed methods are provided in the online version of this paper and include the following:

- KEY RESOURCES TABLE
- EXPERIMENTAL MODEL AND STUDY PARTICIPANT DETAILS
- METHOD DETAILS
  - Tumor mutational burden (TMB) quantification and single-base-substitution (SBS) signature analysis
  - Digital-spatial microscopy (CODEX)
  - Single-cell RNA- and TCR-sequencing and analysis
  - TCR-sequencing and analysis
  - Spectral flow cytometry
  - Cytometry by time of flight (CYTOF)
- QUANTIFICATION AND STATISTICAL ANALYSIS

CellPress
OPEN ACCESS

SUPPLEMENTAL INFORMATION

Supplemental information can be found online at https://doi.org/10.1016/j.xcrm.2026.102889.

Received: December 30, 2025
Revised: April 15, 2026
Accepted: June 5, 2026
Published: June 26, 2026

REFERENCES

1. Velho, P.I., Lim, D., Wang, H., Park, J.C., Kaur, H.B., Almutairi, F., Carducci, M.A., Denmeade, S.R., Markowski, M.C., Isaacs, W.B., et al. (2019). Molecular Characterization and Clinical Outcomes of Primary Gleason Pattern 5 Prostate Cancer After Radical Prostatectomy. JCO Precis. Oncol. *3*, PO.19.00081. https://doi.org/10.1200/PO.19.00081.

2. Zhou, Y., Lin, C., Hu, Z., Yang, C., Zhang, R., Ding, Y., Wang, Z., Tao, S., and Qin, Y. (2021). Differences in survival of prostate cancer Gleason 8-10 disease and the establishment of a new Gleason survival grading system. Cancer Med. *10*, 87–97. https://doi.org/10.1002/cam4.3571.

3. Tsai, A.K., Kagalwalla, S., Langer, J., Le-Kumar, T., Le-Kumar, V., and Antonarakis, E.S. (2024). Pembrolizumab for metastatic castration-resistant prostate cancer: trials and tribulations. Expert Opin. Biol. Ther. *24*, 51–62. https://doi.org/10.1080/14712598.2024.2311750.

4. Lenis, A.T., Ravichandran, V., Brown, S., Alam, S.M., Katims, A., Truong, H., Reisz, P.A., Vasselman, S., Nweji, B., Autio, K.A., et al. (2024). Microsatellite Instability, Tumor Mutational Burden, and Response to Immune Checkpoint Blockade in Patients with Prostate Cancer. Clin. Cancer Res. *30*, 3894–3903. https://doi.org/10.1158/1078-0432.CCR-23-3403.

5. van Wilpe, S., Taha, T., Rothmann, E.C., Altshuler, E., Park, J., Ledet, E.M., Rothermundt, C., Bergman, A.M., Willemsen, A.E.C.A.B., Tsantoulis, P., et al. (2025). Efficacy of Anti-PD-(L)1 Immunotherapy in Patients with DNA Mismatch Repair-deficient Metastatic Castration-resistant Prostate Cancer. Eur. Urol. Oncol. *8*, 1020–1029. https://doi.org/10.1016/j.euo.2025.04.016.

6. Chu, Y., Dai, E., Li, Y., Han, G., Pei, G., Ingram, D.R., Thakkar, K., Qin, J.-J., Dang, M., Le, X., et al. (2023). Pan-cancer T cell atlas links a cellular stress response state to immunotherapy resistance. Nat. Med. *29*, 1550–1562. https://doi.org/10.1038/s41591-023-02371-y.

7. Almeida, J.-S., Casanova, J.M., Santos-Rosa, M., Tarazona, R., Solana, R., and Rodrigues-Santos, P. (2023). Natural Killer T-like Cells: Immunobiology and Role in Disease. Int. J. Mol. Sci. *24*, 2743. https://doi.org/10.3390/ijms24032743.

8. Wang, Q., Chen, S., Guo, Z., Xia, S., and Zhang, M. (2024). NK-like CD8 T cell: one potential evolutionary continuum between adaptive memory and innate immunity. Clin. Exp. Immunol. *217*, 136–150. https://doi.org/10.1093/cei/uxae038.

9. Alam, M.R., Akinyemi, A.O., Wang, J., Howlader, M., Farahani, M.E., Nur, M., Zhang, M., Gu, L., and Li, Z. (2025). CD4+CD8+ double-positive T cells in immune disorders and cancer: Prospects and hurdles in immunotherapy. Autoimmun. Rev. *24*, 103757. https://doi.org/10.1016/j.autrev.2025.103757.

10. Ortolani, C., Forti, E., Radin, E., Cibin, R., and Cossarizza, A. (1993). Cytofluorimetric identification of two populations of double positive (CD4+,CD8+) T lymphocytes in human peripheral blood. Biochem. Biophys. Res. Commun. *191*, 601–609. https://doi.org/10.1006/bbrc.1993.1260.

11. Ager, C.R., Obradovic, A., McCann, P., Chaimowitz, M., Wang, A.L.E., Shaikh, N., Shah, P., Pan, S.S., Laplaca, C.J., Virk, R.K., et al. (2026). Neoadjuvant Fc-enhanced anti-CTLA-4 targets Tregs to augment androgen deprivation in high-risk prostate cancer: A randomized phase I trial. Cell Rep. Med. *7*, 102638. https://doi.org/10.1016/j.xcrm.2026.102638.

12. Singh, R., Kim, Y.-H., Lee, S.-J., Eom, H.-S., and Choi, B.K. (2024). 4-1BB immunotherapy: advances and hurdles. Exp. Mol. Med. *56*, 32–39. https://doi.org/10.1038/s12276-023-01136-4.

13. Simoni, Y., Becht, E., Fehlings, M., Loh, C.Y., Koo, S.-L., Teng, K.W.W., Yeong, J.P.S., Nahar, R., Zhang, T., Kared, H., et al. (2018). Bystander CD8+ T cells are abundant and phenotypically distinct in human tumour infiltrates. Nature *557*, 575–579. https://doi.org/10.1038/s41586-018-0130-2.

14. Chen, Y., Wang, D., Li, Y., Qi, L., Si, W., Bo, Y., Chen, X., Ye, Z., Fan, H., Liu, B., et al. (2024). Spatiotemporal single-cell analysis decodes cellular dynamics underlying different responses to immunotherapy in colorectal cancer. Cancer Cell *42*, 1268–1285.e7. https://doi.org/10.1016/j.ccell.2024.06.009.

15. Chow, R.D., Michaels, T., Bellone, S., Hartwich, T.M.P., Bonazzoli, E., Iwasaki, A., Song, E., and Santin, A.D. (2023). Distinct Mechanisms of Mismatch-Repair Deficiency Delineate Two Modes of Response to Anti-PD-1 Immunotherapy in Endometrial Carcinoma. Cancer Discov. *13*, 312–331. https://doi.org/10.1158/2159-8290.CD-22-0686.

16. Zhang, Y., Chen, H., Mo, H., Hu, X., Gao, R., Zhao, Y., Liu, B., Niu, L., Sun, X., Yu, X., et al. (2021). Single-cell analyses reveal key immune cell subsets associated with response to PD-L1 blockade in triple-negative breast cancer. Cancer Cell *39*, 1578–1593.e8. https://doi.org/10.1016/j.ccell.2021.09.010.

17. Luoma, A.M., Suo, S., Wang, Y., Gunasti, L., Porter, C.B.M., Nabilsi, N., Tadros, J., Ferretti, A.P., Liao, S., Gurer, C., et al. (2022). Tissue-resident memory and circulating T cells are early responders to pre-surgical cancer immunotherapy. Cell *185*, 2918–2935.e29. https://doi.org/10.1016/j.cell.2022.06.018.

18. Cercek, A., Foote, M.B., Rousseau, B., Smith, J.J., Shia, J., Sinopoli, J., Weiss, J., Lumish, M., Temple, L., Patel, M., et al. (2025). Nonoperative Management of Mismatch Repair-Deficient Tumors. N. Engl. J. Med. *392*, 2297–2308. https://doi.org/10.1056/NEJMoa2404512.

19. Guven, H., Gilljam, M., Chambers, B.J., Ljunggren, H.G., Christensson, B., Kimby, E., and Dilber, M.S. (2003). Expansion of natural killer (NK) and natural killer-like T (NKT)-cell populations derived from patients with B-chronic lymphocytic leukemia (B-CLL): a potential source for cellular immunotherapy. Leukemia *17*, 1973–1980. https://doi.org/10.1038/sj.leu.2403083.

20. Bojarska-Junak, A., Hus, I., Sieklucka, M., Wasik-Szczepanek, E., Mazurkiewicz, T., Polak, P., Dmoszynska, A., and Rolinski, J. (2010). Natural killer-like T CD3+/CD16+CD56+ cells in chronic lymphocytic leukemia: intracellular cytokine expression and relationship with clinical outcome. Oncol. Rep. *24*, 803–810. https://doi.org/10.3892/or_00000924.

21. Jadidi-Niaragh, F., Jeddi-Tehrani, M., Ansaripour, B., Razavi, S.M., Sharifian, R.A., and Shokri, F. (2012). Reduced frequency of NKT-like cells in patients with progressive chronic lymphocytic leukemia. Med. Oncol. *29*, 3561–3569. https://doi.org/10.1007/s12032-012-0262-4.

22. Almeida, J.-S., Couceiro, P., López-Sejas, N., Alves, V., Růžičková, L., Tarazona, R., Solana, R., Freitas-Tavares, P., Santos-Rosa, M., and Rodrigues-Santos, P. (2019). NKT-Like (CD3+CD56+) Cells in Chronic Myeloid Leukemia Patients Treated With Tyrosine Kinase Inhibitors. Front. Immunol. *10*, 2493. https://doi.org/10.3389/fimmu.2019.02493.

23. Valhondo, I., Hassouneh, F., Lopez-Sejas, N., Pera, A., Sanchez-Correa, B., Guerrero, B., Bergua, J.M., Arcos, M.J., Bañas, H., Casas-Avilés, I., et al. (2020). Characterization of the DNAM-1, TIGIT and TACTILE Axis on Circulating NK, NKT-Like and T Cell Subsets in Patients with Acute Myeloid Leukemia. Cancers (Basel) *12*, 2171. https://doi.org/10.3390/cancers12082171.

24. Pang, Y.-L., Zhang, H.-G., Peng, J.-R., Pang, X.-W., Yu, S., Xing, Q., Yu, X., Gong, L., Yin, Y.-H., Zhang, Y., and Chen, W.F. (2009). The immunosuppressive tumor microenvironment in hepatocellular carcinoma. Cancer Immunol. Immunother. *58*, 877–886. https://doi.org/10.1007/s00262-008-0603-5.

25. Al Omar, S.Y., Marshall, E., Middleton, D., and Christmas, S.E. (2012). Increased numbers but functional defects of CD56+CD3+ cells in lung cancer. Int. Immunol. *24*, 409–415. https://doi.org/10.1093/intimm/dxr122.
26. Peng, L.-S., Mao, F.-Y., Zhao, Y.-L., Wang, T.-T., Chen, N., Zhang, J.-Y., Cheng, P., Li, W.-H., Lv, Y.-P., Teng, Y.-S., et al. (2016). Altered phenotypic and functional characteristics of CD3+CD56+ NKT-like cells in human gastric cancer. Oncotarget *7*, 55222–55230. https://doi.org/10.18632/oncotarget.10484.
27. Krijgsman, D., de Vries, N.L., Skovbo, A., Andersen, M.N., Swets, M., Bastiaannet, E., Vahrmeijer, A.L., van de Velde, C.J.H., Heemskerk, M.H.M., Hokland, M., and Kuppen, P.J.K. (2019). Characterization of circulating T-NK-and NKT cell subsets in patients with colorectal cancer: the peripheral blood immune cell profile. Cancer Immunol. Immunother. *68*, 1011–1024. https://doi.org/10.1007/s00262-019-02343-7.
28. Bochem, J., Zelba, H., Amaral, T., Spreuer, J., Soffel, D., Eigentler, T., Wagner, N.B., Uslu, U., Terheyden, P., Meier, F., et al. (2019). Peripheral PD-1+CD56+ T-cell frequencies correlate with outcome in stage IV melanoma under PD-1 blockade. PLoS One *14*, e0221301. https://doi.org/10.1371/journal.pone.0221301.
29. Clénet, M.-L., Gagnon, F., Moratalla, A.C., Viel, E.C., and Arbour, N. (2017). Peripheral human CD4+CD8+ T lymphocytes exhibit a memory phenotype and enhanced responses to IL-2, IL-7 and IL-15. Sci. Rep. *7*, 11612. https://doi.org/10.1038/s41598-017-11926-2.
30. Parrot, T., Oger, R., Allard, M., Desfrançois, J., Raingeard de la Blétière, D., Coutolleau, A., Preisser, L., Khammari, A., Dréno, B., Delneste, Y., et al. (2020). Transcriptomic features of tumour-infiltrating CD4lowCD8high double positive αβ T cells in melanoma. Sci. Rep. *10*, 5900. https://doi.org/10.1038/s41598-020-62664-x.
31. Schad, S.E., Chow, A., Mangarin, L., Pan, H., Zhang, J., Ceglia, N., Caushi, J.X., Malandro, N., Zappasodi, R., Gigoux, M., et al. (2022). Tumor-induced double positive T cells display distinct lineage commitment mechanisms and functions. J. Exp. Med. *219*, e20212169. https://doi.org/10.1084/jem.20212169.
32. Menard, L.C., Fischer, P., Kakrecha, B., Linsley, P.S., Wambre, E., Liu, M. C., Rust, B.J., Lee, D., Penhallow, B., Manjarrez Orduno, N., and Nadler, S. G. (2018). Renal Cell Carcinoma (RCC) Tumors Display Large Expansion of Double Positive (DP) CD4+CD8+ T Cells With Expression of Exhaustion Markers. Front. Immunol. *9*, 2728. https://doi.org/10.3389/fimmu.2018.02728.
33. Cercek, A., Lumish, M., Sinopoli, J., Weiss, J., Shia, J., Lamendola-Essel, M., El Dika, I.H., Segal, N., Shcherba, M., Sugarman, R., et al. (2022). PD-1 Blockade in Mismatch Repair-Deficient, Locally Advanced Rectal Cancer. N. Engl. J. Med. *386*, 2363–2376. https://doi.org/10.1056/NEJMoa2201445.
34. Hanssen, F., Garcia, M.U., Folkersen, L., Pedersen, A.S., Lescai, F., Jodoin, S., Miller, E., Seybold, M., Wacker, O., Smith, N., et al. (2024). Scalable and efficient DNA sequencing analysis on different compute infrastructures aiding variant discovery. NAR Genom. Bioinform. *6*, lqae031. https://doi.org/10.1093/nargab/lqae031.
35. Li, H. (2013). Aligning sequence reads, clone sequences and assembly contigs with BWA-MEM. Preprint at arXiv. https://doi.org/10.48550/ARXIV.1303.3997.
36. Auwera, G.V. der, and O'Connor, B.D. (2020). Genomics in the Cloud: Using Docker, GATK, and WDL in Terra, First edition (O'Reilly)).
37. McLaren, W., Gil, L., Hunt, S.E., Riat, H.S., Ritchie, G.R.S., Thormann, A., Flicek, P., and Cunningham, F. (2016). The Ensembl Variant Effect Predictor. Genome Biol. *17*, 122. https://doi.org/10.1186/s13059-016-0974-4.
38. Danecek, P., Bonfield, J.K., Liddle, J., Marshall, J., Ohan, V., Pollard, M.O., Whitwham, A., Keane, T., McCarthy, S.A., Davies, R.M., and Li, H. (2021). Twelve years of SAMtools and BCFtools. GigaScience *10*, giab008. https://doi.org/10.1093/gigascience/giab008.
39. Díaz-Gay, M., Vangara, R., Barnes, M., Wang, X., Islam, S.M.A., Vermes, I., Duke, S., Narasimman, N.B., Yang, T., Jiang, Z., et al. (2023). Assigning mutational signatures to individual samples and individual somatic mutations with SigProfilerAssignment. Bioinformatics *39*, btad756. https://doi.org/10.1093/bioinformatics/btad756.
40. Bankhead, P., Loughrey, M.B., Fernández, J.A., Dombrowski, Y., McArt, D.G., Dunne, P.D., McQuaid, S., Gray, R.T., Murray, L.J., Coleman, H. G., et al. (2017). QuPath: Open source software for digital pathology image analysis. Sci. Rep. *7*, 16878. https://doi.org/10.1038/s41598-017-17204-5.
41. Weigert, M., and Schmidt, U. (2022). Nuclei Instance Segmentation and Classification in Histopathology Images with Stardist. In 2022 IEEE International Symposium on Biomedical Imaging Challenges (ISBIC) (IEEE), pp. 1–4. https://doi.org/10.1109/ISBIC56247.2022.9854534.
42. Stoltzfus, C.R., Filipek, J., Gern, B.H., Olin, B.E., Leal, J.M., Wu, Y., Lyons-Cohen, M.R., Huang, J.Y., Paz-Stoltzfus, C.L., Plumlee, C.R., et al. (2020). CytoMAP: A Spatial Analysis Toolbox Reveals Features of Myeloid Cell Organization in Lymphoid Tissues. Cell Rep. *31*, 107523. https://doi.org/10.1016/j.celrep.2020.107523.
43. Zheng, G.X.Y., Terry, J.M., Belgrader, P., Ryvkin, P., Bent, Z.W., Wilson, R., Ziraldo, S.B., Wheeler, T.D., McDermott, G.P., Zhu, J., et al. (2017). Massively parallel digital transcriptional profiling of single cells. Nat. Commun. *8*, 14049. https://doi.org/10.1038/ncomms14049.
44. Korsunsky, I., Millard, N., Fan, J., Slowikowski, K., Zhang, F., Wei, K., Baglaenko, Y., Brenner, M., Loh, P.r., and Raychaudhuri, S. (2019). Fast, sensitive and accurate integration of single-cell data with Harmony. Nat. Methods *16*, 1289–1296. https://doi.org/10.1038/s41592-019-0619-0.
45. Aran, D., Looney, A.P., Liu, L., Wu, E., Fong, V., Hsu, A., Chak, S., Naikawadi, R.P., Wolters, P.J., Abate, A.R., et al. (2019). Reference-based analysis of lung single-cell sequencing reveals a transitional profibrotic macrophage. Nat. Immunol. *20*, 163–172. https://doi.org/10.1038/s41590-018-0276-y.
46. Hao, Y., Hao, S., Andersen-Nissen, E., Mauck, W.M., Zheng, S., Butler, A., Lee, M.J., Wilk, A.J., Darby, C., Zager, M., et al. (2021). Integrated analysis of multimodal single-cell data. Cell *184*, 3573–3587.e29. https://doi.org/10.1016/j.cell.2021.04.048.
47. Aibar, S., González-Blas, C.B., Moerman, T., Huynh-Thu, V.A., Imrichova, H., Hulselmans, G., Rambow, F., Marine, J.-C., Geurts, P., Aerts, J., et al. (2017). SCENIC: single-cell regulatory network inference and clustering. Nat. Methods *14*, 1083–1086. https://doi.org/10.1038/nmeth.4463.
48. Yang, Q., Safina, K.R., Nguyen, K.D.Q., Tuong, Z.K., and Borcherding, N. (2025). scRepertoire 2: Enhanced and efficient toolkit for single-cell immune profiling. PLoS Comput. Biol. *21*, e1012760. https://doi.org/10.1371/journal.pcbi.1012760.
49. Voong, K.R., Illei, P.B., Presson, B., Singh, D., Zeng, Z., Lanis, M., Hales, R.K., Hu, C., Tran, P.T., Georgiades, C., et al. (2023). Ablative radiation alone in stage I lung cancer produces an adaptive systemic immune response: insights from a prospective stud. J. Immunother. Cancer *11*, e007188. https://doi.org/10.1136/jitc-2023-007188.
50. Popov, A., Samokhina, M., Balashov, I., Nazarov, V.I., bot, immunarch., Rumynskiy, E., Lam, G., and Zarodniuk, M. (2025). immunomind/immunarch: 0.10.3. Version 0.10.3. Zenodo. https://doi.org/10.5281/ZENODO.3367200.
51. Robinson, M.D., McCarthy, D.J., and Smyth, G.K. (2010). edgeR : a Bioconductor package for differential expression analysis of digital gene expression data. Bioinformatics *26*, 139–140. https://doi.org/10.1093/bioinformatics/btp616.
52. McInnes, L., Healy, J., and Melville, J. (2018). UMAP: Uniform Manifold Approximation and Projection for Dimension Reduction. Preprint at arXiv. https://doi.org/10.48550/ARXIV.1802.03426.

STAR★METHODS

KEY RESOURCES TABLE

REAGENT or RESOURCE	SOURCE	IDENTIFIER
Antibodies		
CD34 Atto550	Akoya Biosciences	Cat# 4250057; RRID: AB_3676526
CD56 AF647	Akoya Biosciences	Cat# STP7000
HLA-DR AF750	Akoya Biosciences	Cat# 4450095; RRID: AB_3094500
CD45RO Atto550	Akoya Biosciences	Cat# 4250023; RRID: AB_2895053
FOXP3 AF647	Akoya Biosciences	Cat# 4550071; RRID: AB_2927679
CD20 AF750	Akoya Biosciences	Cat# 4450094; RRID: AB_3094498
CD44 Atto550	Akoya Biosciences	Cat# 4450041; RRID: AB_2936081
IDO1 AF647	Akoya Biosciences	Cat# 4550123; RRID: AB_3476035
Ki-67 AF750	Akoya Biosciences	Cat# 4450096; RRID: AB_3094497
IFNG Atto550	Akoya Biosciences	Cat# 4250062; RRID: AB_3476455
TCF-1 AF647	Akoya Biosciences	Cat# 4550068; RRID: AB_3717468
Pan-Cytokeratin AF750	Akoya Biosciences	Cat# 4450093; RRID: AB_3662772
iNOS Atto550	Akoya Biosciences	Cat# 4250073; RRID: AB_3676531
CD4 AF647	Akoya Biosciences	Cat# 4550112; RRID: AB_3094499
Granzyme-B Atto550	Akoya Biosciences	Cat# 4250055; RRID: AB_3472025
HIF1A AF647	Akoya Biosciences	Cat# 4550069; RRID: AB_3082972
CD8 Atto550	Akoya Biosciences	Cat# 4250012; RRID: AB_2915960
PD-1 AF647	Akoya Biosciences	Cat# 4550038; RRID: AB_3096407
PD-L1 AF647	Akoya Biosciences	Cat# 4550128; RRID: AB_3676534
CD57 Atto550	Akoya Biosciences	Cat# 4250108; RRID: AB_3717470
CD11c AF647	Akoya Biosciences	Cat# 4550135; RRID: AB_3678450
CD163 Atto550	Akoya Biosciences	Cat# STP7000
CD31 Atto550	Akoya Biosciences	Cat# 4250104; RRID: AB_3662762
CD45 AF647	Akoya Biosciences	Cat# 4550121; RRID: AB_3674468
CD66 AF647	Akoya Biosciences	Cat# 4550001; RRID: AB_3475664
CD68 AF647	Akoya Biosciences	Cat# 4550113; RRID: AB_2935894
CD3ε AF647	Akoya Biosciences	Cat# 4550125; RRID: AB_3094503
CD7	Abcam	Cat# ab230834; RRID: AB_2889384
CD3 Spark UV 387	BioLegend	Cat# 323066
CD4 AF680	Invitrogen	Cat# 606-0049-42; RRID: AB_2896239
CD8 APC/Fire-810	BioLegend	Cat# 344764; RRID: AB_2860890
CD11c PE	BD Biosciences	Cat# 566730; RRID: AB_2869833
CD14 BV605	BioLegend	Cat# 301834; RRID: AB_2563798
CD16 BV711	BioLegend	Cat# 302044; RRID: AB_2563802
CD19 APC-H7	BD Biosciences	Cat# 560727; RRID: AB_1727437
CD20 BV786	BD Biosciences	Cat# 568713; RRID: AB_3684489
CD21 BUV496	BD Biosciences	Cat# 750614; RRID: AB_2874746
CD24 BUV395	BD Biosciences	Cat# 563818; RRID: AB_2632389
CD27 PE-CF594	BD Biosciences	Cat# 562297; RRID: AB_11154596
CD36 BV480	BD Biosciences	Cat# 746612; RRID: AB_2871651
CD38 BUV661	BD Biosciences	Cat# 612969; RRID: AB_2870242
CD40 PE-Cy7	BioLegend	Cat# 334322; RRID: AB_10645472
CD45RA BUV563	BD Biosciences	Cat# 612926; RRID: AB_2870211

(Continued on next page)

Continued

REAGENT or RESOURCE	SOURCE	IDENTIFIER
CD56 PE/Fire-810	BioLegend	Cat# 392435; RRID: AB_2927902
CD86 BUV737	BD Biosciences	Cat# 612784; RRID: AB_2814790
CD123 PacBlue	BioLegend	Cat# 306044; RRID: AB_2750165
CD138 PE/Fire-640	BioLegend	Cat# 356550
CCR7 PE/Fire-744	BioLegend	Cat# 353276
CXCR3 APC	BioLegend	Cat# 353707; RRID: AB_10962949
CXCR5 PE-Cy5	BioLegend	Cat# 356952
HLA-DR BV570	BioLegend	Cat# 307638; RRID: AB_2650882
IgD BUV805	BD Biosciences	Cat# 742039; RRID: AB_2871332
IgM AF700	BioLegend	Cat# 314538; RRID: AB_2566615
LOX-1 BV421	BioLegend	Cat# 358610; RRID: AB_2728343
PD-1 BUV615	BD Biosciences	Cat# 612991; RRID: AB_2870262
CD45 89Y	StandardBioTools	Cat# 201349
CD196/CCR6 141Pr	StandardBioTools	Cat# 201349
CD123 143ND	StandardBioTools	Cat# 201349
CD19 144ND	StandardBioTools	Cat# 201349
CD4 145ND	StandardBioTools	Cat# 201349
CD8a 146ND	StandardBioTools	Cat# 201349
CD11c 147Sm	StandardBioTools	Cat# 201349
CD16 148ND	StandardBioTools	Cat# 201349
CD45RO 149Sm	StandardBioTools	Cat# 201349
CD45RA 150ND	StandardBioTools	Cat# 201349
CD161 151Eu	StandardBioTools	Cat# 201349
CD194/CCR4 152Sm	StandardBioTools	Cat# 201349
CD25 153Eu	StandardBioTools	Cat# 201349
CD27 154Sm	StandardBioTools	Cat# 201349
CD57 155Gd	StandardBioTools	Cat# 201349
CD183/CXCR3 156Gd	StandardBioTools	Cat# 201349
CD185/CXCR5 158Gd	StandardBioTools	Cat# 201349
CD28 160Gd	StandardBioTools	Cat# 201349
CD38 161Dy	StandardBioTools	Cat# 201349
CD56/NCAM 163Dy	StandardBioTools	Cat# 201349
TCRgd 164Dy	StandardBioTools	Cat# 201349
CD294 166Er	StandardBioTools	Cat# 201349
CD197/CCR7 167Er	StandardBioTools	Cat# 201349
CD14 168Er	StandardBioTools	Cat# 201349
CD3 170Er	StandardBioTools	Cat# 201349
CD20 171Yb	StandardBioTools	Cat# 201349
CD66b 172Yb	StandardBioTools	Cat# 201349
HLA-DR 173Yb	StandardBioTools	Cat# 201349
IgD 174Yb	StandardBioTools	Cat# 201349
CD127 176Yb	StandardBioTools	Cat# 201349
CD69 113Cd	StandardBioTools	Cat# 3113002B
KLRG1 114Cd*	Biolegend	Cat# 367702; RRID: AB_2632728
CD62L 116Cd*	Biolegend	Cat# 304835; RRID: AB_2563758
FoxP3 159Tb	StandardBioTools	Cat# 3159039A
Ki-67 162Dy	StandardBioTools	Cat# 3162012B
CD279/PD-1 165Ho	StandardBioTools	Cat# 3165042B

(*Continued on next page*)

Continued

REAGENT or RESOURCE	SOURCE	IDENTIFIER
TCF-1 169Tm	Biolegend	Cat# 655202; RRID: AB_2562103
CD278/ICOS 175Lu	StandardBioTools	Cat# 3175039B
Perforin 196Pt	StandardBioTools	Cat# 3196002C
Granzyme-B 198Pt	StandardBioTools	Cat# 3198002C
TIGIT 209Bi	StandardBioTools	Cat# 3209013B
Deposited data		
Raw and analyzed data	This paper	GEO: GSE332846
Software and algorithms		
nf-core/sarek v3.4.4	Menard et al.[29]	https://github.com/nf-core/sarek/tree/3.4.4
Burrows-Wheeler Aligner v0.7.18	Li[30]	https://github.com/lh3/bwa
Genome Analysis Toolkit v4.5.0.0	Auwera and O'Connor[31]	https://github.com/broadinstitute/gatk
Mutect2 v4.2.6.1	Auwera and O'Connor[31]	https://github.com/TRON-Bioinformatics/tronflow-mutect2
GATK HaplotypeCaller v4.2.6.1	Auwera and O'Connor[31]	https://github.com/broadinstitute/gatk
Ensembl Variant Effect Predictor v110	McLaren et al.[32]	https://github.com/Ensembl/ensembl-vep
Bcftools v1.21	Danecek et al.[33]	https://github.com/samtools/bcftools
SigProfilerAssignment v1.0.3	Díaz-Gay et al.[34]	https://github.com/AlexandrovLab/SigProfilerAssignment
QuPath v0.4.4	Bankhead et al.[35]	https://qupath.github.io/
Stardist v0.9.1	Weigert and Schmidt[36]	https://github.com/stardist/stardist
cytoMAP v1.4.21	Stoltzfus et al.[37]	https://gitlab.com/gernerlab/cytomap
Cell Ranger v9.0.0	Zheng et al.[38]	https://github.com/10XGenomics/cellranger
Seurat v4.3.0	Hao et al.[39]	https://satijalab.org/seurat/
Harmony v1.2.0	Korsunsky et al.[40]	https://github.com/immunogenomics/harmony
SingleR v1.4.1	Aran et al.[41]	https://github.com/dviraran/SingleR
AUCell v1.24.0	Aibar et al.[42]	https://github.com/aertslab/AUCell
scRepertoire v2.3.4	Yang et al.[43]	https://github.com/BorchLab/scRepertoire
Immunarch R package v0.10.3	Popov et al.[44]	https://github.com/immunomind/immunarch
edgeR	Robinson et al.[45]	https://github.com/OliverVoogd/edgeR
Pheatmap v1.0.13	N/A	https://github.com/raivokolde/pheatmap
OMIQ	Dotmatrics	https://www.omiq.ai/
JMP v19	JMP	https://www.jmp.com/en/home
FlowJo v9	BD Biosciences	https://www.flowjo.com/
Maxpar Pathsetter	Standard Biotools	https://www.standardbio.com/products/software/maxpar-pathsetter
GraphPad Prism v10	Dotmatics	https://www.graphpad.com/features

EXPERIMENTAL MODEL AND STUDY PARTICIPANT DETAILS

The 67-year-old otherwise healthy Caucasian male patient provided written informed consent for collection and biobanking of blood samples along with interrogation of tissue samples according to an internal review board (IRB)-approved protocol (IRB #00003639). The patient's age and gender did not influence the results of the study.

METHOD DETAILS

Tumor mutational burden (TMB) quantification and single-base-substitution (SBS) signature analysis

Total DNA was isolated from formalin-fixed, paraffin-embedded (FFPE) tumor tissue that was microdissected for tumor purity, as well as paired peripheral blood. Samples were sequenced using the Novaseq 6000 whole-exome sequencer alongside a panel designed

to enrich for >20,000 genes to detect amplifications and deletions. Paired-end reads for each sample were processed using the nf-core/sarek workflow (v3.4.4).[34] Reads were aligned to the human GRCh38 reference genome using the Burrows-Wheeler Aligner (v0.7.18).[35] The Genome Analysis Toolkit (GATK, v4.5.0.0)[36] was used to identify the number of unique, duplicate, and unmapped read pairs from the aligned file. Mutect2 (v4.2.6.1)[36] and GATK HaplotypeCaller (v4.2.6.1)[36] were used to detect somatic and germline variants, respectively, in the tumor-normal pair. Ensembl Variant Effect Predictor (VEP, v110)[37] was then used to predict the consequences of the variants on gene and protein sequences. Bcftools (v1.21)[38] was implemented to filter somatic variants to those with variant allele frequencies (VAFs) $\geq$0.01, total read depth $\geq$50 reads, and allelic depth $\geq$3 reads. Similar thresholds were implemented for germline variants, except for a VAF cutoff. All variants were filtered for a variant effect predictor (VEP) consequence of 'missense' or greater severity and variants without a gene symbol were omitted. To estimate tumor mutational burden (TMB), the total number of variants were divided by 25 megabases, given the total exon region size for the whole-exome sequencing panel. Lastly, SigProfilerAssignment[39] was applied to assign and visualize Catalog of Somatic Mutations in Cancer (COSMIC) signatures for the tumor-normal pair using the somatic and germline variants detected. A de-identified dataset of real-world patients' samples that underwent comprehensive molecular profiling at a CLIA/CAP-certified lab (Caris Life Sciences) was queried for TMB values in prostate cancer patients. Next-generation sequencing (NGS) was performed on genomic DNA isolated from FFPE tumor samples using NextSeq or NovaSeq 6000 platforms. TMB values were compiled from patients with histology-confirmed prostate cancer and were stratified by MMR and MSI status.

Digital-spatial microscopy (CODEX)

Five-micron histological sections were cut from the FFPE biopsy and prostatectomy samples, mounted onto a slide and stained according to the manufacturer's protocol for imaging on the Phenocycler Fusion 2.0 (Akoya Biosciences). The sections were deparaffinized and rehydrated, followed by antigen retrieval with citrate buffer (Fisher Scientific; Cat# 00-4955-58). All antibodies (see key resources table) were purchased conjugated to DNA barcodes except anti-human CD7 (Abcam; Cat# ab230834), which was conjugated to a DNA barcode (Akoya Biosciences; Cat# 5550024) using an antibody conjugation kit (Akoya Biosciences; Cat# 7000009). After the slide was stained with primary antibodies, autofluorescence removal was performed in a light bath consisting of 4.5% hydrogen peroxide and 27 mM sodium hydroxide in phosphate-buffered saline (PBS) for two rounds of 45 min. Imaging was performed at 20$\times$ on a Phenocycler Fusion 2.0 (Akoya Biosciences). Complementary barcodes conjugated to Atto550, Alexa Fluor 647 and Alexa Fluor 750 were used in cyclical hybridization, buffer exchange, imaging and stripping cycles. Image stitching, deconvolution, alignment and cycle concatenation were performed in Akoya Biosciences software. Images were examined and further analyzed in QuPATH software (v0.4.4).[40] Antibodies had previously been validated on a human tonsil sample, but any markers that produced a low signal-to-noise ratio or untenable staining pattern at this stage were rejected (CD16, CD45RA, CD49a, CD69, CD11b). Tissue regions that exhibited poor imaging quality were excluded from subsequent analysis. Nuclei were segmented using Stardist[41] and a neural network classifier was trained to identify positive cells for each channel. Unsupervised clustering of regions was performed in cytoMAP[42] on cell objects extracted from QuPATH. Raster scanning was used to define neighborhoods and these were clustered into self-organizing maps with region number defined by the Davies-Bouldin criterion, normalizing mean fluorescence intensity to max mean fluorescence intensity for all cells, inputting the nearest neighbor self-organizing map, and clustering by markers of interest including the mean membrane intensity for CD45, CD3ε, CD4, CD8, CD56, CD7, CD11c, PD-1, and nuclear intensity for Ki-67. For image quantification, T cells are defined as $CD45^+CD3\varepsilon^+$ cells and further subdivided by other markers (CD4, CD8, CD45RO, PD-1, Ki-67).

Single-cell RNA- and TCR-sequencing and analysis

Cryopreserved peripheral blood mononuclear cells (PBMCs) were thawed and adjusted to a final concentration of 1,000–2,000 viable cells/μL. Approximately 17,000 cells were loaded onto the Chromium X (10$\times$ Genomics) to capture a target of 10,000 cells per sample. Libraries were prepared using the Chromium Next GEM Single Cell V(D)J Reagent Kits v1.1, following the manufacturer's instructions. The 5′ digital gene expression (DGE) libraries were sequenced on an Illumina NovaSeq X system to a target depth of 50,000 reads per cell, while full-length V(D)J segments were enriched from barcoded cDNA to enable pairing of TCRα and TCRβ chains with the corresponding transcriptome. The 5′ V(D)J libraries were sequenced on an Illumina NovaSeq X system to a depth of approximately 5,000 reads per cell.

For data pre-processing, Cell Ranger (v.9.0.0)[43] was used to align reads to the human GRCh38 human reference genome, assign cell barcodes, and generate count matrices. Low-quality cells were filtered based on total number of unique genes detected (<200) and proportion of mitochondrial genes (>20%). Data was normalized using the SCTransform function in the Seurat pipeline (v4.3.0).[46] The top 2,000 highly variable genes (HVGs) were used for principal component analysis and Harmony (v1.2.0)[44] was implemented to correct batch effects. Seurat was used to apply Uniform Manifold Approximation and Projection (UMAP) for dimensionality reduction and unsupervised clustering.

SingleR (v1.4.1)[45] was used in conjunction with Blueprint/ENCODE reference transcriptomes to annotate immune cell types within the samples. To comprehensively annotate T/NK cell subsets, $CD8^+$ T cells, $CD4^+$ T cells, and NK cells were isolated and differentially expressed genes (DEGs) were calculated across clusters. DEGs were then manually inspected for markers of T cell and NK cell phenotypes.

CellPress
OPEN ACCESS

To infer the functional potential of each distinct T/NK cell subset, we employed AUCell (v1.24.0)[47] which applies rank-based scoring to infer the enrichment of gene sets within single cells. Functional gene sets were based on previously published signatures.[6]

scV(D)J-seq data was analyzed using the Cell Ranger VDJ pipeline which aligned V(D)J-seq reads to GRCh38 to assemble TCR sequences. scRepertoire (v2.3.4)[48] was then implemented to combine TCR information with the single-cell transcriptomes.

For dMMR/MSI-H colorectal,[14] dMMR/MSI-H endometrial carcinoma,[15] triple-negative breast cancer,[16] and head/neck squamous cell carcinoma[17] validation datasets, count matrices aligned and mapped to the GRCh38 human reference genome were acquired. Similarly, low quality cells were filtered, and data was normalized using the SCTransform function in the Seurat pipeline. Harmony was implemented to correct batch effects and UMAP was applied for dimensionality reduction and unsupervised clustering. T cells were annotated using SingleR. Fold expansion was calculated using the percentage of T cell subtype out of all cells, comparing post-versus pre-ICI proportions.

TCR-sequencing and analysis

TCR-sequencing was performed on FFPE prostate biopsies and PBMCs across four timepoints by the Johns Hopkins Sidney Kimmel Comprehensive Cancer Center FEST and TCR Immunogenomics Core as previously described.[49] Briefly, DNA was extracted from PBMCs using the DNeasy Blood and Tissue Kit (Qiagen). The TCR-beta locus was amplified and sequenced using the AmpliSeq for Illumina TCR beta-short read assay. Data preprocessing was performed to eliminate non-productive TCR sequences and to align and trim the nucleotide sequences to obtain only the complementarity-determining region 3 (CDR3). Sequences not beginning with C or ending with F or W and having less than seven amino acids in the CDR3 were eliminated. Processed data was analyzed using the Immunarch R package[50] for computing basic clonotype statistics and visualizing the processed TCR-seq data, in which raw clonotype counts were normalized by library size in counts per million (CPM) with edgeR.[51] Individual clonotypes with CPM $\geq$5 were retained as this threshold efficiently excluded outliers without affecting the mean distribution. Within each sample, clonotypes were ranked by CPM and top 40 most expanded clonotypes were selected. Sample-specific top clonotype lists were merged based on the CDR3 amino acid sequences to create a clonotype-by-sample matrix. Missing values in any sample were assigned a CPM value of 0 to indicate absence. CPM values were then log-transformed, and the final matrix was visualized as a heatmap using the pheatmap package (v1.0.13).

Spectral flow cytometry

Immunophenotyping was performed on cryopreserved PBMCs using spectral flow cytometry. Briefly, PBMCs were thawed using a CryoThaw device (Medax; Cat# 1-9X-NIH1) in a thawing medium composed of RPMI-1640, 10% heat-inactivated FBS, and 0.02 mg/mL DNase I (Stemcell; Cat# 07900). The thawed cells were centrifuged at 300g for 8 min, and the resulting cell pellet was washed with thawing medium and then with PBS.

To assess cell viability and prevent non-specific antibody binding, the cells were resuspended in PBS and incubated with LIVE/DEAD Fixable Blue Dead Cell Stain (Invitrogen; Cat# L23105; 1:1000 dilution) and Human Fc Block (BD Biosciences; Cat# 564219; 1:20 dilution) for 20 min at room temperature, protected from light. Following a wash with PBS, the cells were stained with a pre-titrated surface antibody cocktail (see key resources table) diluted in a staining buffer consisting of PBS with 50% v/v BD Horizon Brilliant Stain Buffer (BD Biosciences; Cat# 563794). This incubation was performed for 20 min at room temperature, protected from light. After staining, the samples were washed twice with PBS and subsequently fixed in 1% paraformaldehyde (PFA) in PBS for 15 min at room temperature. Finally, the cells were washed and resuspended in PBS for data acquisition on a 4-laser Cytek Aurora spectral flow cytometer. Unmixed FCS files were then analyzed using OMIQ (Dotmatics) or FlowJo (BD Biosciences; v10). Principal component analysis (PCA) plots were produced using JMP (v19). For UMAP dimensional analysis, 35,000 events from each timepoint were concatenated and analyzed using the UMAP plugin (v4.1.1)[52] within FlowJo.

Cytometry by time of flight (CYTOF)

Cryopreserved human PBMCs were thawed and rested overnight at 37°C prior to staining with the Maxpar Direct Immune Profiling Assay (MDIPA; Standard BioTools; Cat# 201349). Up to 3×10^6 cells per timepoint were prepared for staining via incubation with an Fc receptor blocking solution (Biolegend; Cat# 422301) and then transferred to an MDIPA tube for surface staining. Additional surface antibodies (see key resources table), including Live/dead 103Rh (StandardBioTools; Cat# 201349) were added by hand. After surface staining, samples were washed and processed following the MDIPA protocol for PBMCs with minimal edits for intracellular staining (see key resources table). Samples were prepared for intracellular staining using the FoxP3/transcription factor staining buffer set (eBioscience; Cat# 00–5523). After intracellular staining, samples were fixed with 1.6% fresh formaldehyde and held overnight at 4°C in Maxpar Fix/Perm with DNA intercalator. Stained samples were acquired on the HeliosTM mass cytometer with EQ four element calibration beads (Standard BioTools; Cat# 201078). The resulting files were normalized and cleaned to isolate single-cell events. Basic analysis was completed within the Maxpar Pathsetter analysis platform to identify lymphocyte subsets or via Flowjo (BD Biosciences; v10). Metal-tagged antibodies not commercially available were conjugated using the Maxpar antibody labeling kit (Standard Biotools) and stored in antibody stabilizer or HRP protector (Boca Scientific; Cat# 131 000 or 222 000). For PC validation datasets,[11] patient CyTOF samples with $\geq$10,000 CD45$^+$ events were concatenated and analyzed using the UMAP plugin within FlowJo.

QUANTIFICATION AND STATISTICAL ANALYSIS

Variance normality in all datasets was tested using Shapiro-Wilk tests. Parametric ANOVA with Tukey or nonparametric Kruskal-Wallis tests were used when comparing >2 groups. Parametric unpaired t-tests or nonparametric Mann-Whitney tests were used when comparing two groups. Wilcoxen tests were used for T cell signature score comparisons from scRNA-seq results. Statistical significance for all tests was defined as *$p < 0.05$, **$p < 0.01$, ***$p < 0.001$, and ****$p < 0.0001$. Statistics were performed in Prism (v10).

Cell Reports Medicine, Volume *7*

Supplemental information

Case of complete response to immunotherapy in MMR-deficient prostate cancer associated with NK-like and CD4^{+}CD8^{+} T cells

Alexander K. Tsai, John R. Lozada, Philippa R. Kennedy, David Moline, Rachana Pandey, Riley C. Lyons, Christine Luo, Rulin Wang, Ali T. Arafa, Elise L. Femino, Sarah Zipkowitz, Alexis Figueroa, Patrick J. McCann, Matthew C. Dallos, Andrew Elliott, Paari Murugan, Martin Felices, Nicholas A. Zorko, Badrinath R. Konety, Scott M. Dehm, Jeffrey S. Miller, Steven S. Shen, Elizabeth A. Thompson, Laura A. Sena, Srinivasan Yegnasubramanian, Justin Hwang, and Emmanuel S. Antonarakis

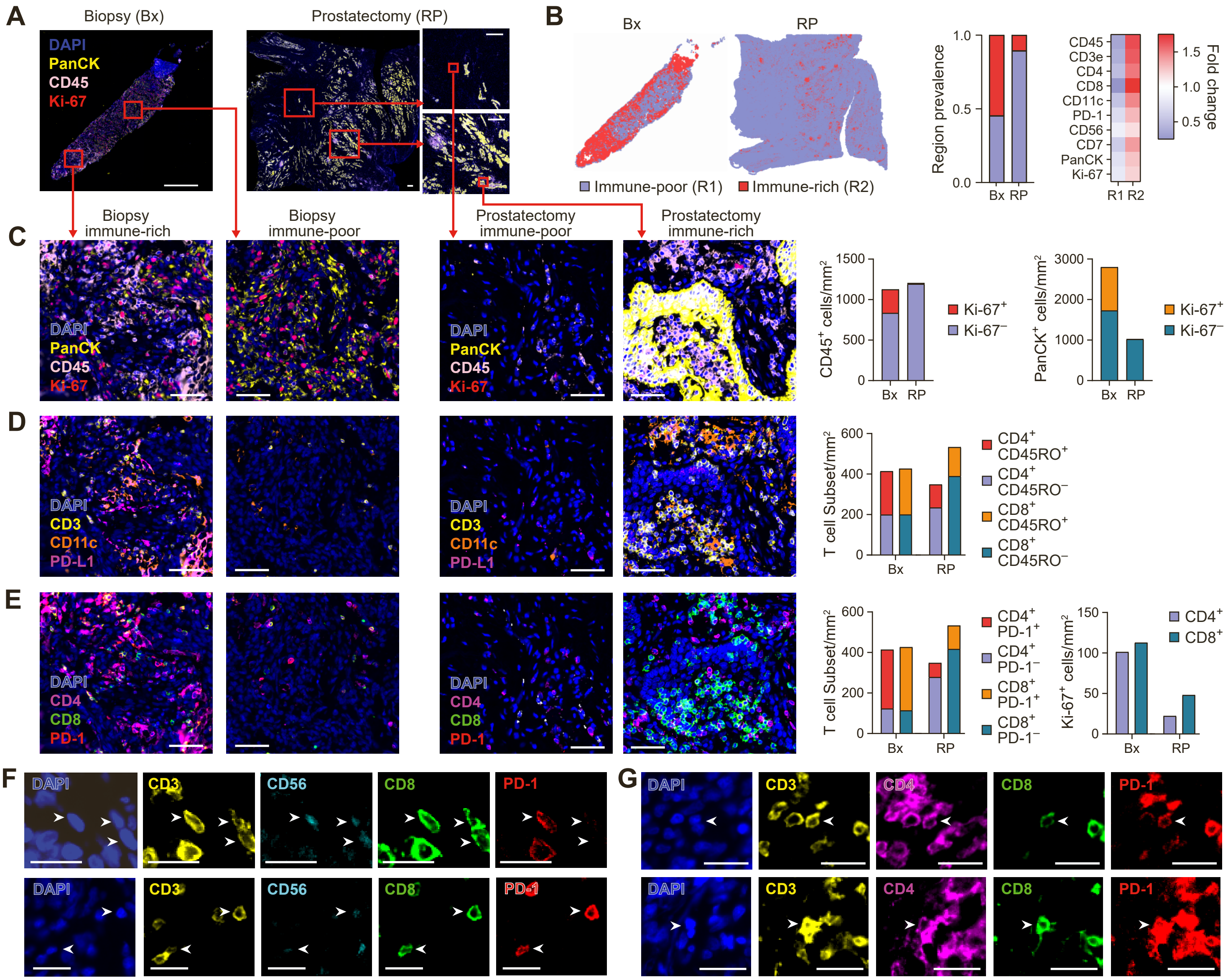

Figure S1. Antigen-experienced T cells were enriched in pre-ICI prostate cancer biopsies. Related to Figures 1 and 2. Multiplex tissue imaging of formalin-fixed, paraffin-embedded (FFPE) biopsy (Bx; pre-treatment) and prostatectomy (RP; post-ICI). All channels are scaled consistently apart from pancytokeratin (A, C) which is dimmer on tumor cells (biopsy) than glands (prostatectomy). Consequently, two different PanCK thresholds were applied to biopsy and prostatectomy samples to visualize tumor position. Quantifications of cell populations in (C-E) are shown in right panels and were performed in QuPATH.

(A) Representative channels and regions from biopsy and prostatectomy specimens. Insets show areas used for representative images in further panels. Scale bars, 0.5 mm.

(B) Unsupervised region determination by cytoMAP software depicting immune-poor (R1) and immune-rich (R2) regions in biopsies and prostatectomy tissue. Quantifications are shown in the middle panel while fold changes for individual markers between R1 and R2 regions are shown in the right panel.

(C) Representative images (left) depict PanCK^{+} tumor cells (biopsy) and glandular cells (prostatectomy), CD45^{+} leukocytes, and proliferating Ki-67^{+} cells. Scale bars, 50 µm. Graphs (right) quantify Ki-67^{+} proliferating CD45^{+} leukocytes and PanCK^{+} tumor or glandular cells.

(D) Representative images (left) depict CD3^{+} T cells, CD11c^{+} cells, and expression of PD-L1. Scale bars, 50 µm. Graph (right) quantifies CD45RO^{+} (antigen-experienced) CD4^{+} and CD8^{+} T cells.

(E) Representative images (left) depict CD4^{+} and CD8^{+} T cells and PD-1 expression. Scale bars, 50 µm. Graphs (right) quantify PD-1^{+} (antigen-experienced and possibly exhausted) and Ki-67^{+} (proliferating) CD4^{+} and CD8^{+} T cells.

(F) Representative CD3^{+}CD8^{+}CD56^{+} "NK-like" T cells, indicated with white arrows. Scale bars, 20 µm.

(G) Representative CD3^{+}CD4^{+}CD8^{+} "double-positive" (DP) T cells, indicated with white arrowheads. Scale bars, 20 µm.

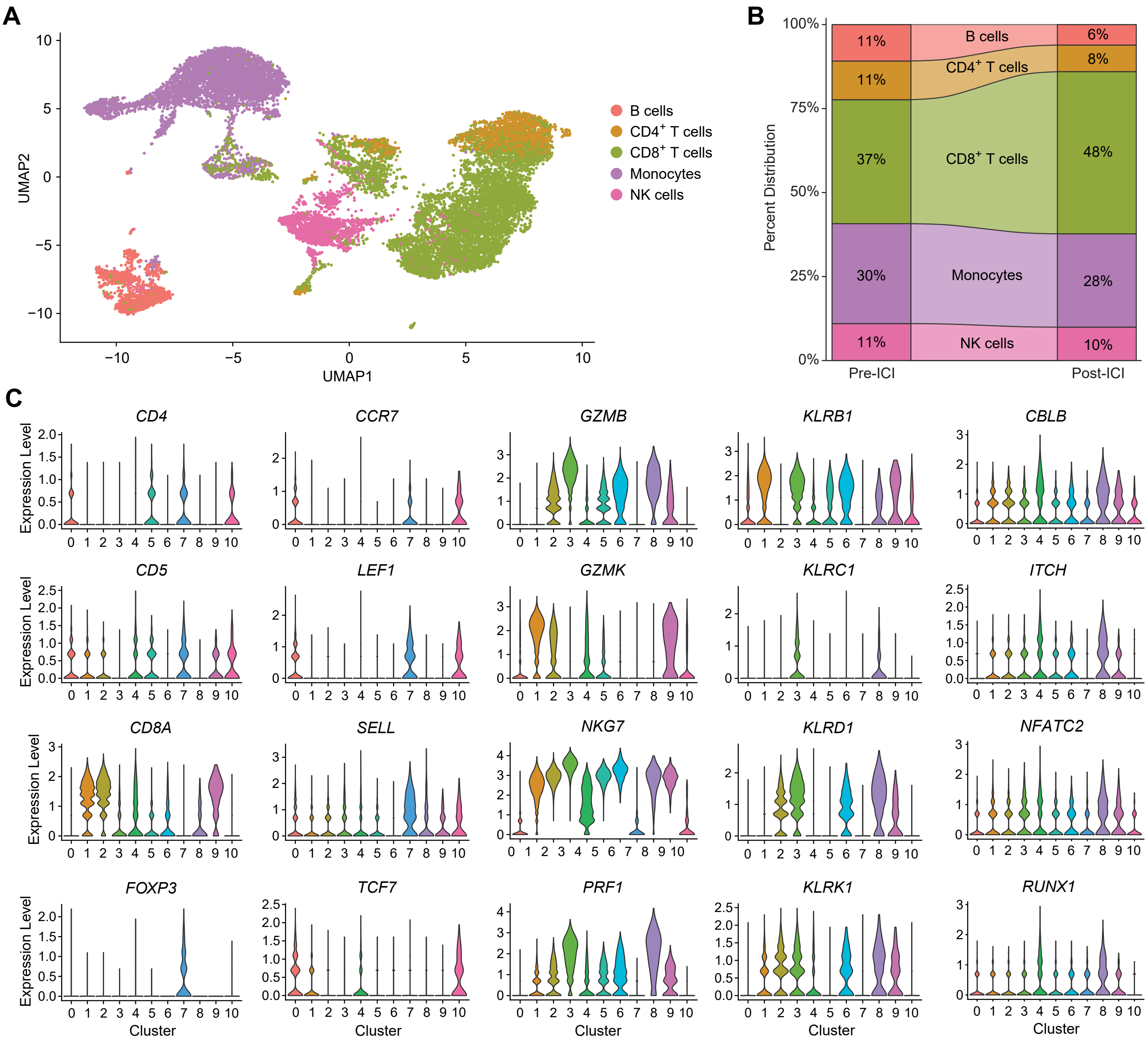

Figure S2. NK/T cell cluster annotation. Related to Figure 2.

(A) Clustering of PBMCs compiled from single-cell RNA-sequencing performed using pre-ICI and post-ICI timepoint #1 identifying B cells, $CD4^+$ T cells, $CD8^+$ T cells, monocytes, and NK cells.

(B) Distribution of cell subsets in (A) at pre-ICI and post-ICI timepoints.

(C) Expression of T cell genes (*CD4*, *CD5*, *CD8A*, *FOXP3*), memory/naïve T cells (*CCR7*, *LEF1*, *SELL*, *TCF7*), T cell effector genes (*GZMB*, *GZMK*, *NKG7*, *PRF1*), NK-associated genes (*KLRB1*, *KLRC1*, *KLRD1*, *KLRK1*), and anergy-associated genes (*CBLB*, *ITCH*, *NFATC2*, *RUNX1*) within all NK/T cell clusters (see Figure 2A).

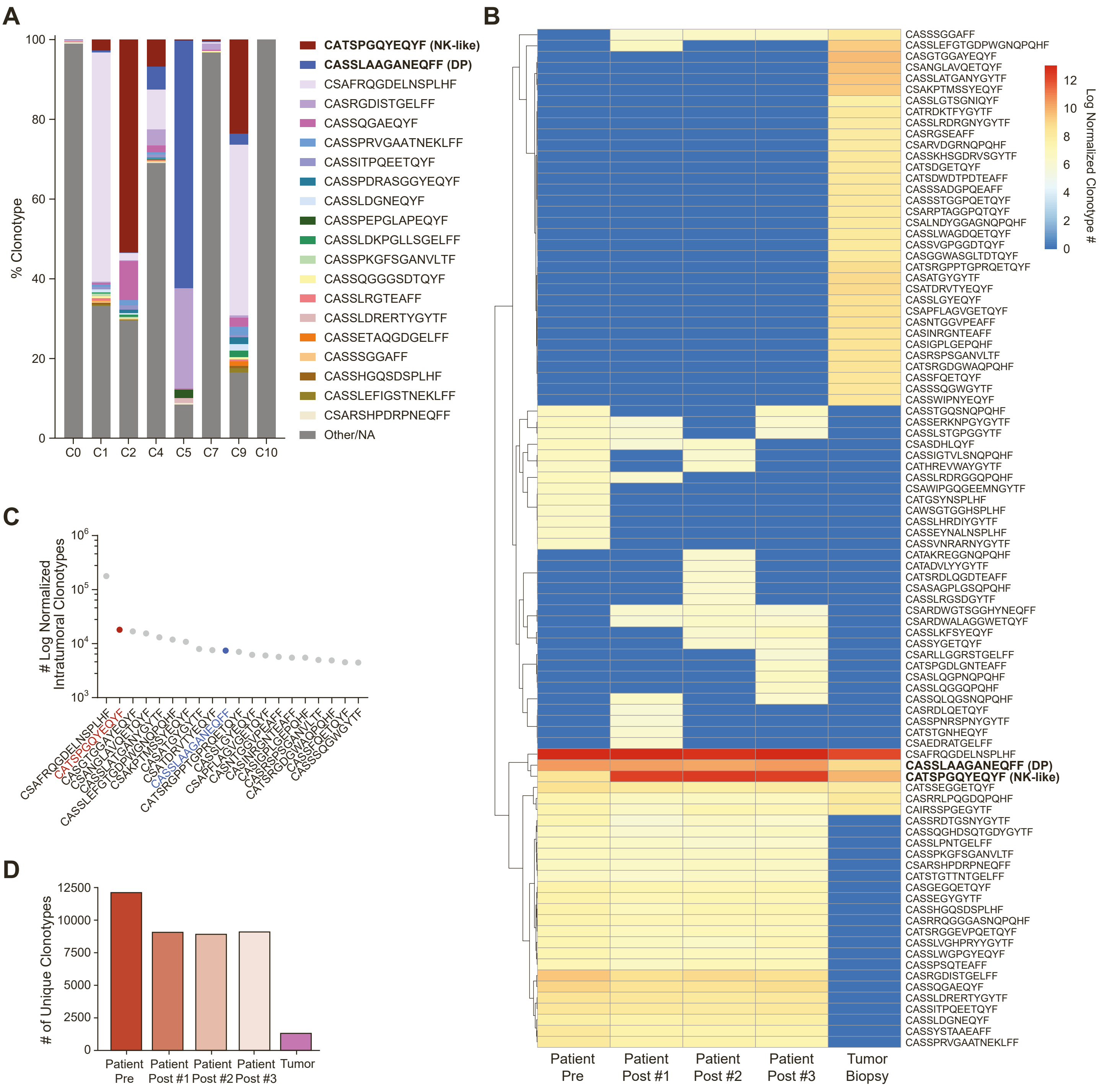

Figure S3. Intratumoral and peripheral TCR repertoire. Related to Figure 2.
(A) Frequency of the most abundant 20 TCR clonotypes within each T cell cluster for combined PBMC Pre and Post #1 timepoints as assessed by scTCR-seq. The designation "Other/NA" includes all other TCR clonotypes and cells lacking TCR data.
(B) TCR clonotypes detected within circulation and in pre-treatment tumor biopsy tissue as assessed by bulk TCR-sequencing. The top 40 most abundant log-transformed clonotypes from each of the five samples are shown after count-per-million (cpm) normalization and filtering.
(C) Log-transformed normalized clonotype count in tumor biopsy tissue by bulk TCR-sequencing.
(D) Number of unique clonotypes detected within circulation and in pre-treatment tumor biopsy tissue by bulk TCR-sequencing.

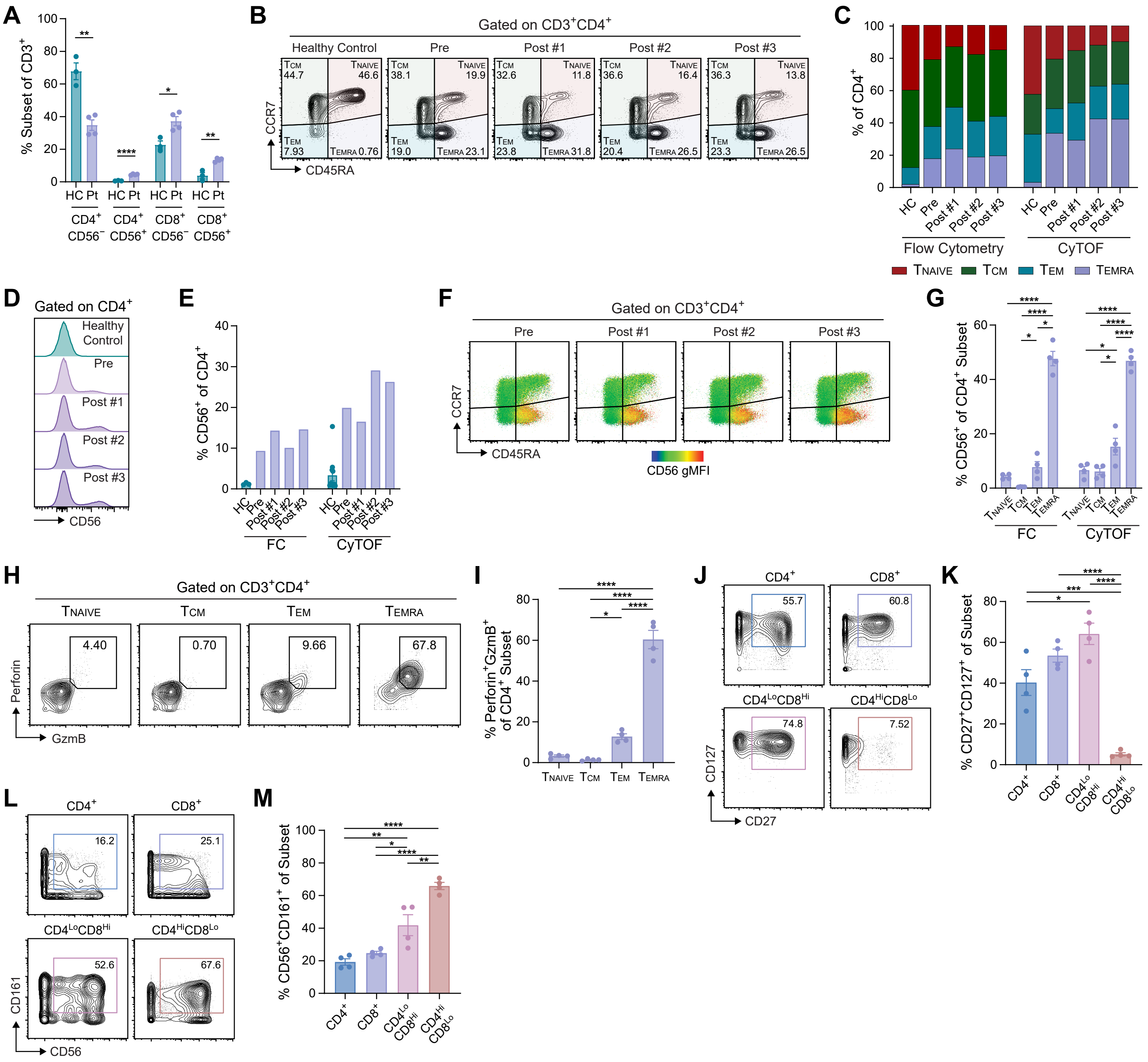

Figure S4. NK-like T cells differentiate into TEMRA and exhibited cytotoxic potential. Related to Figure 3.

(A) CD4^{+}CD56^{-}, CD4^{+}CD56^{+}, CD8^{+}CD56^{-}, and CD8^{+}CD56^{+} frequencies within the T cell (CD45^{+}CD3^{+}) compartment of healthy control (HC) and patient PBMCs at all four timepoints assessed by flow cytometry. Unpaired t-tests were used for comparisons with statistical significance denoted as * p<0.05, ** p<0.01, and **** p<0.0001.

(B-C) Representative plots (B) of frequencies of TNAIVE (CCR7^{+}CD45RA^{+}), TCM (CCR7^{+}CD45RA^{-}), TEM (CCR7^{-}CD45RA^{-}), and TEMRA (CCR7^{-}CD45RA^{+}) within the CD4^{+} T cell compartment of HC and patient PBMCs (C) assessed by flow cytometry and CyTOF.

(D) Histograms of CD56 on CD4^{+} T cells from flow cytometry showing a representative HC and the patient at all four timepoints.

(E) CD56^{+} frequencies of CD4^{+} T cells from patient and HC PBMCs assessed by flow cytometry and CyTOF.

(F-G) Heatmaps of CD56 geometric mean fluorescent intensity (gMFI; F) with CD56^{+} frequencies based on TNAIVE, TCM, TEM, and TEMRA CD4^{+} T cell subsets (combined across all four timepoints; G) assessed by flow cytometry and CyTOF. ANOVA with Tukey tests were used for comparisons with statistical significance denoted as * p<0.05 and **** p<0.0001.

(H-I) Representative plots (H) of perforin^{+}granzyme-B^{+} (GzmB^{+}) frequencies of TNAIVE, TCM, TEM, and TEMRA CD4^{+} T cell subsets (combined across all four timepoints; I) assessed by CyTOF. ANOVA with Tukey tests were used for comparisons with statistical significance denoted as * p<0.05 and **** p<0.0001.

(J-K) Representative plots (J) of memory-associated CD27^{+}CD127^{+} cells within CD4^{+}, CD8^{+}, CD4^{Lo}CD8^{Hi}, and CD4^{Hi}CD8^{Lo} patient-derived T cells (K) assessed by CyTOF. ANOVA with Tukey tests were used for comparisons with statistical significance denoted as * p<0.05, *** p<0.001, and **** p<0.0001.

(L-M) Representative plots (L) of NK-associated CD56^{+}CD161^{+} cells within CD4^{+}, CD8^{+}, CD4^{Lo}CD8^{Hi}, and CD4^{Hi}CD8^{Lo} patient-derived T cells (M) assessed by CyTOF. ANOVA with Tukey tests were used for comparisons with statistical significance denoted as * p<0.05, ** p<0.01, and **** p<0.0001.

All panels show mean ± SEM.

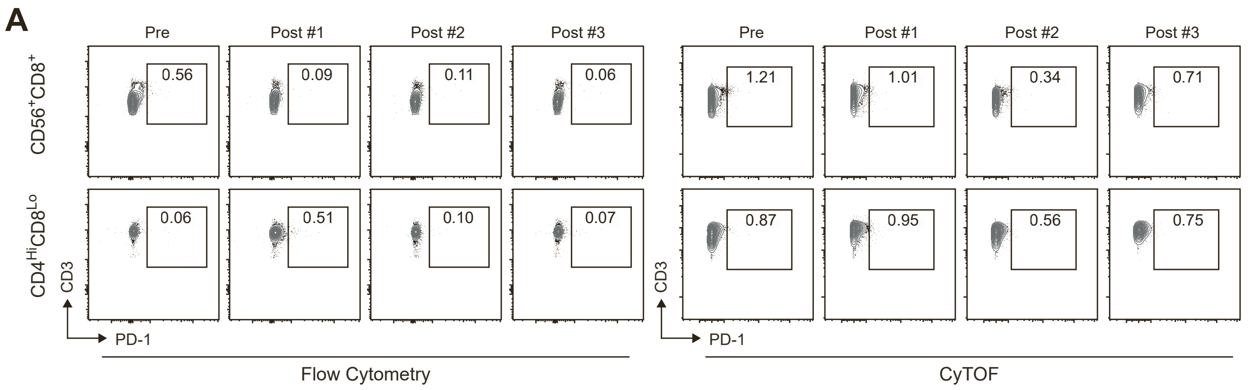

Figure S5. Circulating NK-like and DP T cells lack PD-1. Related to Figure 3.
(A) Plots from spectral flow cytometry (left) and CyTOF (right) of PD-1^{+} frequency of either CD56^{+}CD8^{+} NK-like T cells (upper) or CD4^{Hi}CD8^{Lo} DP T cells (lower) from all four PBMC timepoints.

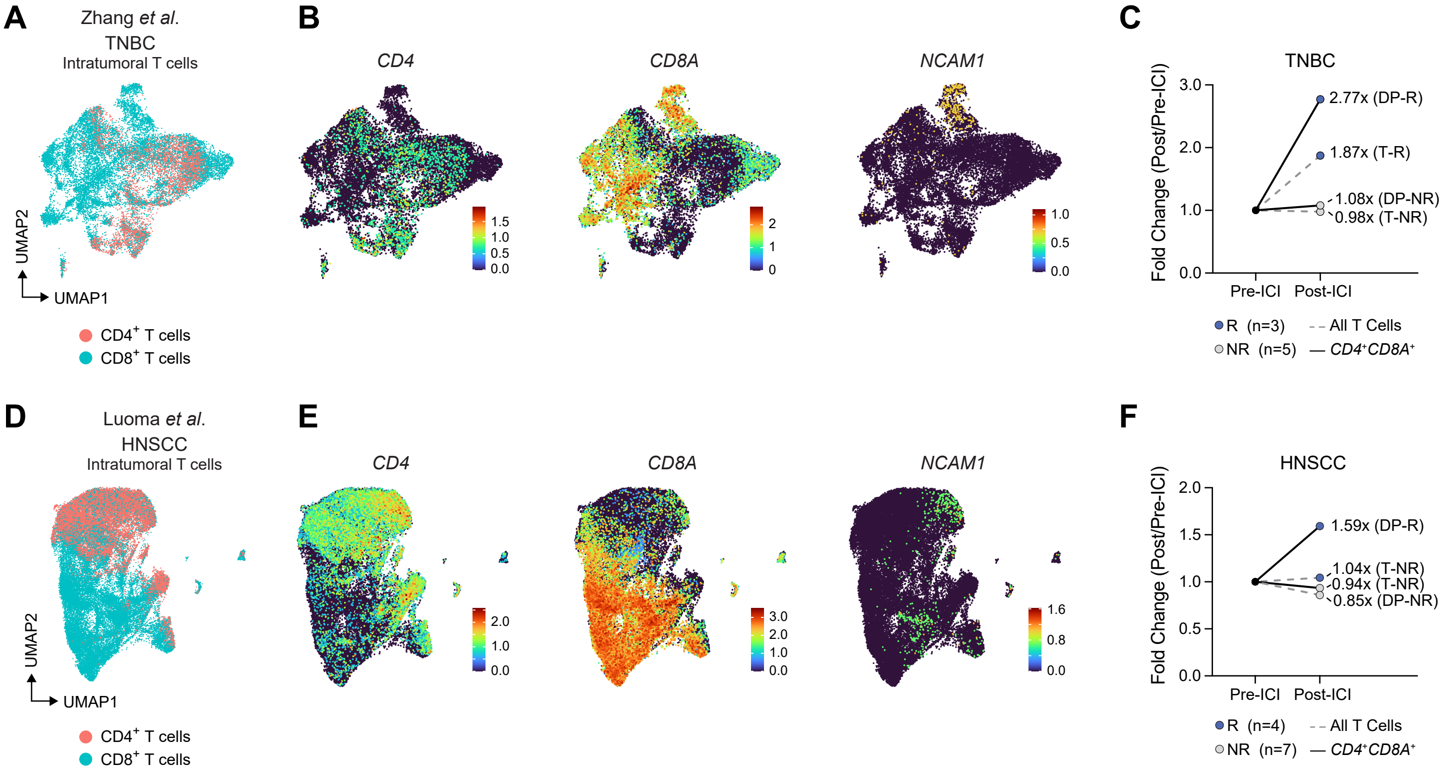

Figure S6. CD4⁺CD8⁺ T cells expand in TNBC and HNSCC ICI responders. Related to Figure 4.
(A) T cell annotation from scRNA-seq of intratumoral T cells in triple-negative breast cancer (TNBC) patients treated with paclitaxel + atezolizumab (see reference 16).
(B) *CD4*, *CD8A*, and *NCAM1* gene expression in TNBC patients.
(C) Fold change in *CD4⁺CD8A⁺* double-positive T cells and all T cells segregated by responders (R) and nonresponders (NR).
(D) T cell annotation from scRNA-seq of intratumoral T cells in head/neck squamous cell carcinoma (HSNCC) patients treated with neoadjuvant nivolumab ± ipilimumab (see reference 17).
(E) *CD4*, *CD8A*, and *NCAM1* gene expression in HNSCC patients.
(F) Fold change in *CD4⁺CD8A⁺* double-positive T cells and all T cells segregated by responders (R) and nonresponders (NR).
